# Efficacy and safety of laparoscopic liver resection versus radiofrequency ablation in patients with early and small hepatocellular carcinoma: an updated meta-analysis and meta-regression of observational studies

**DOI:** 10.1186/s12957-023-03292-3

**Published:** 2024-02-07

**Authors:** Mahmoud Shaaban Abdelgalil, Basma Ehab Amer, Noha Yasen, Mohamed El-Samahy, Ahmed K. Awad, Bahaa Elfakharany, Omar Saeed, Mohamed Abd-ElGawad

**Affiliations:** 1 Faculty of Medicine, Ain-Shams University, Cairo, Egypt; 2 Faculty of Medicine, Benha University, Benha, Egypt; 3 Faculty of Applied Medical Sciences, Misr University for Science and Technology, Cairo, Egypt; 4 Faculty of Medicine, Zagazig University, Zagazig, Egypt; 5 Faculty of Medicine, Ain-Shams University, Cairo, Egypt; 6 Faculty of Allied Medical Sciences, Pharos University, Alexandria, Egypt; 7 Faculty of Medicine, Fayoum University, Fayoum, Egypt; 8 Medical Research Group of Egypt, Negida Academy, Arlington, MA USA

**Keywords:** Hepatocellular carcinoma, Laparoscopic liver resection, Radiofrequency ablation, Overall survival, Recurrence rates

## Abstract

**Background:**

Hepatocellular carcinoma (HCC) is the most common type of liver cancer, accounting for 90% of cases worldwide and a significant contributor to cancer-related deaths. This study comprehensively compares the safety and efficacy of laparoscopic liver resection (LLR) versus laparoscopic or percutaneous radiofrequency ablation (LRFA or PRFA) in patients with early and small HCC.

**Methods:**

We systematically searched Cochrane Library, PubMed, Scopus, and Web of Science databases to include studies comparing LLR versus LRFA or PRFA in patients with early HCC meets the Milan criteria (defined as solitary nodule < 5 cm or three nodules ≤ 3 cm with no extrahepatic spread or vascular invasion). Pooled results were examined for overall survival, disease-free survival, recurrence-free survival, local, intrahepatic and extrahepatic recurrence rates, and complications. We conducted subgroup analyses based on the type of RFA. Meta-regression analyzed the association between overall survival, local recurrence, and various factors. The quality of the included studies was assessed using the Newcastle–Ottawa Scale. We analyzed the data using the R (v.4.3.0) programming language and the “meta” package of RStudio software.

**Results:**

We included 19 observational studies, compromising 3756 patients. LLR showed higher 5-year overall survival compared to RFA (RR = 1.17, 95% CI [1.06, 1.3], *P* > 0.01). Our subgroup analysis showed that LLR had higher 5-year survival than PRFA (RR = 1.15, 95% CI [1.02, 1.31], *P* = 0.03); however, there was no significant difference between LLR and LRFA (RR = 1.26, 95% CI [0.98, 1.63], *P* = 0.07). LLR was associated with higher disease-free survival) RR = 1.19, 95% CI [1.05, 1.35], *P* < 0.01; RR = 1.61, 95% CI [1.31, 1.98], *P* < 0.01(and recurrence-free survival) RR = 1.21, 95% CI [1.09, 1.35], *P* < 0.01; RR = 1.45, 95% CI [1.15, 1.84], *P* < 0.01(at 1 and 3 years. LLR was associated with lower local (RR = 0.28, 95% CI [0.16, 0.47], *P* < 0.01) and intrahepatic recurrence (RR = 0.7, 95% CI [0.5, 0.97], *P* = 0.03) than RFA. However, complications were significantly higher with LLR (RR = 2.01, 95% CI [1.51, 2.68], *P* < 0.01). Our meta-regression analysis showed that younger patients had higher risk for local recurrence (*P* = 0.008), while age wasn’t significantly linked to overall survival (*P* = 0.25). Other covariates like total bilirubin, alpha-fetoprotein levels, and tumor size also showed no significant associations with either overall survival or local recurrence.

**Conclusion:**

LLR offers improved long-term outcomes and lower recurrence rates than PRFA. However, no significant distinctions were observed between LRFA and LLR in overall survival, recurrence-free survival, and local recurrence. More robust well-designed RCTs are essential to validate our findings.

**Supplementary Information:**

The online version contains supplementary material available at 10.1186/s12957-023-03292-3.

## Introduction

Liver cancer poses a global health challenge, with expanding incidence worldwide. It is expected that one million individuals annually will face liver cancer by 2025 [1]. Hepatocellular carcinoma (HCC) dominates, accounting for 90% of cases of liver cancers. It is the fifth most common cancer worldwide and the second major cause of cancer-related deaths due to its aggressiveness [2]. In East Asia and Africa, HCC exhibits notable prevalence and mortality rates, with China at the forefront, housing 466,000 HCC patients – accounting for 55% of global cases – among the yearly count of 854,000 new cases [3, 4]. Additionally, the emergence of increasing cases is evident in various regions of Europe and the USA [1].

Chronic liver disease is the predominant cause of HCC, contributing to 90% of cases. Cirrhosis is the most significant risk factor for HCC, regardless of its etiology. HCC is now the leading cause of death in cirrhotic patients, with an annual occurrence rate of 1–6%. HCC risk factors involve persistent alcohol use, diabetes, and non-alcoholic steatohepatitis related to obesity and HBV or HCV infection [1].

The Barcelona Clinic Liver Cancer (BCLC) algorithm outlines diverse treatment choices for HCC, spanning liver transplantation, surgical resection, and ablation [5]. Due to donor scarcity, liver transplantation is seldom the primary choice. In addition, the effectiveness of surgery and ablation remains a topic of ongoing discussion.

Open hepatic resection is a key curative approach for HCC; however, it presents certain risks and can negatively impact liver function. As a result, this method may not be ideal for patients with severe cirrhosis [6]. Radiofrequency ablation (RFA) emerges as an alternative for small HCC cases, noted for its minimally invasive nature and simplicity. In fact, only 30% of HCC patients are considered good candidates for hepatic resection, underscoring the importance of RFA. Studies indicate that RFA produces comparable outcomes to open resection but with shorter hospital stays and fewer complications. Therefore, both RFA and hepatectomy are recommended for treating early-stage HCC [7].

Recent developments in laparoscopic technology expand the treatment options for HCC, with laparoscopic liver resection (LLR) and laparoscopic radiofrequency ablation (LRFA) gaining traction, especially for cases with small HCC. LLR combines the strengths of RFA and open resection to reduce recurrence risks [8]. While percutaneous RFA is widely used for early-stage HCC, its limitations arise from tumor visibility and positioning. LRFA offers a solution for challenging cases, like subcapsular tumors, where percutaneous methods face difficulties. Previous research emphasizes LRFA's effectiveness and safety for subcapsular HCCs [9–13].

The debate over the most effective and safe treatment for hepatocellular carcinoma is ongoing [9, 14, 15]. Based on previous research, there is a recognized need for a comprehensive assessment of the effectiveness and safety of LLR, LRFA, and PRFA in patients with early HCC. While previous meta-analyses [16–19] have made valuable contributions, they have been limited in study numbers and scope, potentially missing essential insights. For example, Mou‐Bo Si et al. [16], Shan Jin et al. [17], and Xiaocheng Li et al. [20] included 6, 7, and 10 studies, respectively. In contrast, Zhijun Li et al. [19] adopted a more focused approach, scrutinizing Chinese literature and solely including studies from China, with a total of 19 articles (3 in English and 16 in Chinese). However, new studies have emerged in the English literature, providing an opportunity to bolster the impact of the meta-analysis. Surprisingly, previous meta-analyses have yet to concentrate on comparing LLR and LRFA.

Given the advancements in medical knowledge and techniques, an updated systematic review and meta-analysis is essential. This updated analysis aims to fill crucial gaps by directly comparing LLR and laparoscopic/percutaneous RFA and giving the medical community scientifically informed insights to facilitate enhanced clinical decision-making.

## Methods

Our methodology and findings followed systematic review and meta-analysis guidelines, including PRISMA 2020 [21] and the Cochrane Handbook [22]. Transparency was ensured by registering our protocol on PROSPERO with reference “CRD42023436948.”

### Literature search

We performed an extensive search across various databases, including the Cochrane Library, PubMed, Web of Science, and Scopus. Our search spanned from the databases' earliest records to July 31, 2023. We used the following key terms: laparoscopic liver resection, radiofrequency ablation, and hepatocellular carcinoma. We provide our detailed search strategy in the Supplementary file.

### Eligibility criteria and study selection

Two authors (B.E. and N.Y.) screened the article to determine their eligibility for our study focusing on RCTs, non-randomized comparative studies, and observational studies (prospective and retrospective cohorts). Initial screening involved titles and abstracts, followed by a detailed review of chosen study texts.

We included studies comparing LLR versus RFA (percutaneous or laparoscopic) in patients with early-stage HCC meets the Milan criteria (defined as solitary nodule < 5 cm or three nodules ≤ 3 cm with no extrahepatic spread or vascular invasion) [23] or meets University of California San Francisco criteria (defined as a solitary tumor smaller than 6.5 cm or up to three nodules, each less than 4.5 cm in diameter) [24]. Furthermore, eligible patients should exhibit liver function classified as Child–Pugh class A or B (less than 10% fall into the Child–Pugh class C).

Our primary investigation centered on direct comparisons of clinical effectiveness, evaluating parameters such as overall survival, recurrence-free survival rate, disease-free survival rate, local recurrence, intrahepatic recurrence, and extrahepatic recurrence. In terms of safety assessments, we examined the overall incidence of all complications, major complications rated as grade 3 or above, 90-day mortality, 30-day mortality, as well as hospital stay duration. Discrepancies were resolved by a third author.

### Exclusion criteria

We excluded case series, case reports, editorials, cross-sectional and non-human studies, and. Moreover, studies exploring alternative treatments like trans-arterial chemoembolization and percutaneous ethanol injection were excluded. Finally, non-English studies and those with unreliable data were also excluded.

### Quality assessment

Two independents’ authors (B.E and M.E) assessed the quality of included studies using the Newcastle–Ottawa Scale (NOS) [25], which covers the following domains selection, comparability, and outcomes. A top score of 9 is possible, with 7 or higher indicating high quality. Discrepancies were resolved through discussion or involving a third reviewer if necessary.

### Data extraction and study outcomes

Two authors (N.Y AND B. E) used standardized method for data extraction in a predefined Excel sheet, covering study characteristics, patient descriptions, and outcomes of interest. Disagreements were resolved through discussion or consultation with the senior author. Pertinent data were gathered in a predefined Excel sheet, covering study characteristics, patient descriptions, and LLR and RFA outcomes for safety and efficacy. If any study reported their outcomes at different time points, we extracted the data at each timepoint separately, aiming to perform subgroup analysis to explore the change of this outcome overtime.

### Outcome definition

This study rigorously assessed the effectiveness and safety of treatments, employing a comprehensive range of metrics. These measures encompassed overall survival (from treatment onset to death or latest follow-up), recurrence-free survival rate (proportion of patients without HCC recurrence), disease-free survival rate (proportion without disease), hospital stay (duration of patient admission for treatment and recovery), major (grade 3 or above) complications (complications significantly impacting postoperative progress, necessitating interventions), local recurrence (tumor reappearance within liver or nearby original site), intrahepatic recurrence (new tumor nodules or growth within liver separate from primary tumor or previously treated lesions), and extrahepatic recurrence (spread to distant organs).

### Data synthesis and heterogeneity assessment

We conducted our analysis using the R (v.4.3.0) programming language and the “meta” package of RStudio software [26]. We computed the risk ratio (RR) for dichotomous outcomes using the “metabin” function; however, the “metacont” function was used to pool the standardized mean difference (SMD) for continuous outcomes. Given the substantial heterogeneity among the included studies, we preferred to use the random-effects model. We used the 95% confidence intervals (CI) for all outcomes. A *p*-value < 0.05 indicated significance; however, a chi-square *P* value < 0.10 indicated significant heterogeneity among the included studies. We performed subgroup analysis based on the time point of outcome assessment (i.e., at 1, 3, or 5 years). Also, we performed another subgroup analysis based on type of RFA (i.e., LRFA versus PRFA). In addition, we performed sensitivity analyses using the leave-one-out model to explore the effect of each individual study on our results. To assess publication bias, we employed funnel plots, Egger’s test, and trim-and-fill analysis [27]. Finally, we conducted meta-regression analyses to explore whether there was any significant association between the local recurrence and overall survival at 1 year with continuous covariates, such as the age, tumor size, total bilirubin, and alpha-fetoprotein [28].

## Results

### Literature search results

**Fig. 1:**
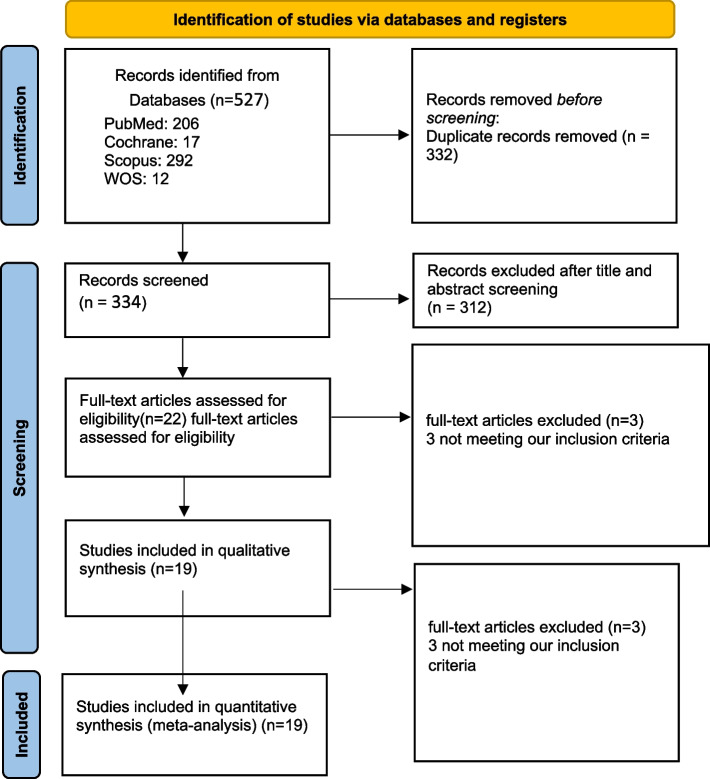
PRISMA flow diagram for included studies

Our comprehensive search yielded 527 records. After removal of duplicates, only 334 records remained for the title and abstract screening. After which, 22 articles seemed eligible for the full-text screening. Finally, we included 19 observational studies in our systematic review and meta-analysis. Reviewing the reference list of all included studies did not retrieve any additional eligible studies. The PRISMA flow diagram is shown in Fig. 1.

### Characteristics of individual studies

Our meta-analysis included 19 observational studies [15, 29–46], compromising 3756 patients. Of which, only one study was prospective [44], while all remaining studies were retrospective [15, 29–43, 45, 46]. The included studies were conducted in five different countries: China (*n* = 7), Japan (*n* = 4), Korea (*n* = 3), Italy (*n* = 3), and Taiwan (*n* = 2). The follow-up duration ranged from one year in Wu 2020 [44] to about 17 years in Cheng 2022 [43]. All included studies used percutaneous RFA, except for Casaccia 2017 [15], Santambrogio 2017 [33], Tsukamoto 2019 [31], and Ko 2022 [39], which used laparoscopic RFA. According to the NOS, the quality of included studies ranged from six to nine points, indicating good to fair quality and low risk of bias in the included studies. Only one study scored nine [32]; however, 12 studies scored eight [15, 29, 31, 33–37, 39, 40, 42, 44, 45], four studies scored seven [30, 38, 41, 43], and three studies scored six [36, 46]. We summarized the included studies and their patients’ baseline characteristics in Table 1 and Supplementary Table 1 respectively.

**Table 1:** Comprehensive overview of the included Studies

Study ID	Country, Study design, Study duration	Sample size of each group	Inclusion Criteria	Type of Resection, The surgical method	Type of RFA	Conclusion	Complications	NOS SCORE
song 2015 [30]	China, retrospective study,2.6 years	LLR (*n* =78) RFA (*n*=78)	• Age: 18–75 years • Definitive diagnosis of primary liver cancer • Single liver tumor: Diameter <4 cm, no vein invasion, lymph node or other metastasis • Liver function: Child–Pugh grade A or B, indocyanine green retention rate <30%at 15 min, platelet count >50 x 10^9/L, thrombin time <5 s • No history of prior treatments: trans arterial chemoembolization, surgery, chemotherapy, or other anti−tumor treatments • Eastern Cooperative Oncology Group score >2	Laparoscopic, 50%were anatomical liver resection	PRFA	There was no difference between LH and RFA in terms of OS in patients with a single, small HCC	Not reported	9
LAI 2016 [36]	China, retrospective study,3 years	LLR (*n*=28), RFA (*n*=33), or OH (*n*=33)	• First diagnosed with HCC and completed treatments at the hospital • Maximum tumor diameter <3 cm, or single lesion <5 cm • Number of intrahepatic tumors≤3 • Child–Pugh class A or B • BCLC stage 0 or A • No intrahepatic or distant metastases • No invasion of specified veins • Indocyanine green retention rate <30%at 15 min • Suitable for LH, RFA, or OH according to guidelines	Laparoscopic, the surgical method not reported	PRFA	Laparoscopic hepatectomy is more effective than PRFA for small HCC, showing similar results to OH but with less trauma. LH is preferred for those under 60, while older patients can choose surgery or PRFA	Not reported	7
Harada2016 [38]	Japan, retrospective study PSM,6 years	LLR group (*n* =81), RFA group (*n* =40)	• Tumors≤3 cm in size, max three tumors or solitary tumor≤5 cm • Diagnosis of portal hypertension (PHT) required presence of EVs and/or platelet count <100,000/µL with splenomegaly • EV presence determined preoperatively via upper gastrointestinal endoscopy • Splenomegaly defined as spleen length >10 cm on preoperative CT	Laparoscopic, 25%were anatomical liver resection	PRFA	By reducing postoperative complications, LR may be a treatment option for patients with BCLC stage 0 or A HCC and PHT	Shoulder pain, ascites superficial surgical site, infection, colitis cholangitis, delirium , bile leakage, pneumonia, atrial fibrillation, deep vein thrombosis, urinary tract infection, pleural effusion, deep surgical site infection, liver failure	8
Casaccia 2017 [15]	Italy, retrospective study,6 years	LLR(*n*=24), LRFA (*n*=22)	• Patients evaluated for liver disease severity using Child–Pugh classification • Plasma levels of alfa−fetoprotein (AFP) measured	Laparoscopic, Most resections were anatomic	LRFA	Initial findings show hepatic resection's superiority over thermoablation for laparoscopic treatment of selected small HCC cases. LLR outperformed LRFA in terms of OS. Larger studies are needed to validate these results	Not reported	8
Santambrogio 2017 [31]	Italy, retrospective study,5 years	LLR (*n* =59), LRFA (*n* =205)	• Study focused on HCC patients treated with LLR or RFA from 1998 to 2017 • Inclusion criteria: • Single lesion • Tumor size < 3 cm • Good liver function (Child–Pugh class A) • < 2 segments resected • Treated once with LRFA or LLR • Comorbidities assessed using Charlson's index • Treatment decisions guided by BCLC staging and tumor location	Laparoscopic, 51%were anatomical liver resection	LRFA	Our data favor hepatic resection for single nodules and good liver function. Thermoablation is suitable for complex cases or poor prognosis, allowing a less invasive approach.	Abdominal wall hematoma, ascites, mild acute encephalopathy, hemoperitoneum, jaundice, transient renal failure, other complications	8
Yamashita 2018 [27]	Japan, retrospective study,10 years	LLR (*n*=38), RFA (*n*=62)	• Primary HCC within the Milan criteria	Laparoscopic, LLR were anatomical and non−anatomical resections	PRFA or LRFA	In severe cirrhosis, multimodal RFA for HCC offers less invasiveness, shorter hospital stays, and maintains patient survival. Consider rethinking the standard treatment for primary HCC within Milan criteria to include multimodal RFA for severe cirrhosis cases	Not reported	8
Tsukamoto 2019 [29]	Japan, retrospective study,8.3 years	LLR (*n*=77), LRFA (*n*=94)	• HCC within the Milan criteria	Laparoscopic, the surgical method not reported	LRFA	For patients with severely impaired liver function, consider E-RFA as a suitable initial treatment for HCC. However, avoid using E-pHR as the primary treatment in these cases	Not reported	8
Chong 2019 [39]	China, retrospective study PSM,12 years	LLR (*n* =59) RFA (*n* =155)	• Patients underwent curative liver resection or RFA for primary HCC • Minimally invasive approach: laparoscopic, robotic hepatectomy, percutaneous, or laparoscopic RFA • BCLC stage 0/A • Resection for subcapsular, solitary, or oligonodular tumors with good liver function and sufficient liver remnant • RFA for cirrhotic patients with small/deep tumors, especially if percutaneous approach feasible • Patient preferences considered if both treatments suitable	laparoscopic or robotic, the surgical method not reported	PRFA or LRFA	In early-stage HCC, MIH offered improved long-term survival compared to RFA, without added complications. When possible, MIH should be prioritized as the primary treatment for these patients	Not reported	7
Pan 2019 [32]	China, retrospective study PSM,3.3 years	LLR (*n* =163), RFA (*n* =314)	• Initial HCC diagnosis via histology or noninvasive AASLD criteria • Solitary tumor≤5.0 cm or multiple tumors (≤3), each≤3.0 cm • Visible lesions on ultrasound with safe path for percutaneous treatment • No extrahepatic metastasis, confirmed by enhanced CT or MRI • Child–Pugh class A or B • Eastern Cooperative Oncology Group performance status of 0	Laparoscopic, 37.4%were anatomical liver resection	PRFA	In early-stage HCC patients, MIH yielded superior long-term survival compared to RFA, without raising complication rates. When possible, MIH should be considered as the preferred initial treatment for this patient group	Allergic shock, postoperative heart failure, postoperative respiratory failure, ascites, pain, fever, vomiting	8
Lee 2020 [44]	Korea, retrospective study,6.6 years	LLR (*n* =251), *p*−RFA (*n* =315)	• Single nodular HCCs≤3 cm • Treated with LLR (laparoscopic liver resection) or p−RFA (percutaneous radiofrequency ablation) • No prior treatment for HCC • No macrovascular invasion or extrahepatic metastasis • Child–Pugh class A liver function • Absence of significant co−existing medical conditions, except HCC	Laparoscopic, the surgical method not reported	PRFA	For small single HCCs located subcapsularly, perivascularly, and anteroinferolaterally, LLR can offer notably improved local tumor control compared to PRFA As such, LLR may be the preferred treatment option	Not reported	6
Lin 2020 [35]	Taiwan, retrospective study,5 years	LLR (*n*=36), RFA (*n*=39)	• Single subcapsular HCC≤2 cm in diameter • Child–Pugh class A liver cirrhosis • Primary treatment with percutaneous CT−or ultrasound−guided RFA or minimally invasive surgery (MIS), including laparoscopic or robotic−assisted approaches	MIS, including laparoscopic or robotic, the surgical method not reported	PRFA	Among patients with single subcapsular HCC (≤ 2 cm), Child–Pugh A liver function, and no significant portal hypertension, demonstrated superior 7-year OS, RF), and DFS compared to PRFA	Not reported	8
Ogiso 2020 [33]	Japan, retrospective study,5 years	LLR(*n*=85), RFA (*n*=136)	• BCLC stage 0 or A • Tumor size≤3 cm • Up to 3 nodules • No macrovascular involvement • Child Pugh class A or B	Laparoscopic, the surgical method not reported	PRFA	RFA is less invasive, although both LLR and RFA are safe and effective. LLR provides better local control with superior recurrence−free and local−recurrence free survival. These results help optimize treatment selection based on patient−specific factors	Not reported	8
Wu 2020 [42]	China, Prospective study,1 year	LLR (*n* =35), RFA (*n* =20)	• Early−stage HCC defined as per specific criteria: • BCLC Stage 0 or A • Tumor size≤3 cm • Up to 3 nodules • No macroscopic vascular invasion or extrahepatic spread • Child–Pugh class A or B	Laparoscopic, the surgical method not reported	PRFA	Ablation is a safe and cheap way to treat PHC at an early stage for its wonderful performance in the postoperative short-term outcome	Not reported	8
Xu 2021 [28]	China, retrospective study,2 years	LLR group (*n*=48), RFA group (*n*=46)	• Single tumor≤6 cm diameter • Diagnosis confirmed by multiple exams (ultrasound, CT, MRI, or puncture) • Patients not in decompensated cirrhosis stage • No invasion of portal vein, hepatic arteriovenous, or inferior vena cava • No metastasis outside the liver • Patients underwent LH (likely laparoscopic hepatectomy) or RFA	Laparoscopic, LLR were anatomical hepatectomy	PRFA	RFA and LH have similar effects in the treatment of small HCC. And RFA has the advantages of less trauma, shorter operation duration, and quick postoperative recovery	Abdominal infection, bleeding, biliary fistula pleural effusion	7
Kim 2021 [43]	Korea, retrospective study PSM,10 years	LLR (*n* =101) RFA (*n* =264)	• Single tumor≤4 cm diameter • No metastasis or vascular invasion • New diagnosis without prior surgical resection or non−surgical HCC treatment • HCC located in AL segments (II, III, IVb, V, and VI)	Laparoscopic, 59%were anatomical liver resection	PRFA	For patients with a single, small HCC located in the anterolateral segments of the liver, LLR was associated with similar complication and overall survival rates, but better disease−free survival compared with RFA. LLR may be recommended for patients with higher α−fetoprotein levels	Organ injury, fluid collection, urinary complication, pulmonary complication, skin burn, others	8
Conticchio 2021 [40]	Italy, retrospective study PSM,3 years	RFA (*n*=98), LLR (*n*=86)	• Child–Pugh class A and B • Age≤70 years • Single hepatocellular carcinoma≤3 cm diameter • No major portal/hepatic vein branch invasion • No extrahepatic disease	Laparoscopic, anatomical and non− anatomical liver resection	PRFA or LRFA	Despite a longer length of hospital stay and operative time, LLR guarantees a comparable postoperative course and a better OS and DFS in elderly patients with single HCC ( 3 cm), located in anterolateral segments	Liver failure, ascites, biliary leakage, hemorrhage, systemic infection, intra−abdominal abscess, wound infection, portal thrombosis, pulmonary, cardiac, renal	8
Cheng 2022 [41]	China, retrospective study, 16.6−years	LLR (*n*=99), RFA (*n*=31)	• Patients underwent RFA or LLR for small HCC • Small HCC defined by: • BCLC stage 0 or A • Size≤3 cm • Up to 3 nodules on CT scan or MRI • No macrovascular invasion	Laparoscopic,45.5%were anatomical liver resection	PRFA	Both RFA and LLR are safe and feasible treatment options for patients with small HCC. LLR should be considered for patients with preserved liver function with a better DFS; while RFA offered a comparable OS with less surgical trauma and shorter hospital stay	Not reported	7
Ko 2022 [37]	Korea, retrospective study PSM,6 years	LRFA (*n* =29), LLR (*n* =60)	• solitary subcapsular HCC between 1 and 3 cm	Laparoscopic, 58.3%were anatomical liver resection	LRFA	There was no significant difference in therapeutic outcomes between LHR and LRFA for single subcapsular HCCs measuring 1–3 cm. The difference in RFS should be further evaluated in a larger study	Not reported	8
Liu 2022 [34]	Taiwan, retrospective study PSM,5 years	LLR, (*n* =119) RFA, (*n* =481)	• BCLC Stage 0 or A • Tumor size≤3 cm • Up to 3 nodules • No macroscopic vascular invasion or extrahepatic spread • Child–Pugh class A or B	Laparoscopic and robotic, the surgical method not reported	PRFA	After PSM, severe postoperative complication and OS rates were found to be comparable between the MIS and RFA groups, but RFS was higher in the MIS group than the RFA group, suggesting that MIS may have better outcomes for patients with early-stage HCC	Not reported	6

*Abbreviations*: *HCC* Hepatocellular carcinoma, *BCLC* Barcelona Clinic Liver Cancer staging system, *LH* Laparoscopic Hepatectomy, *RFA* Radiofrequency Ablation, *PRFA* Percutaneous Radiofrequency Ablation, *OH* Open Hepatectomy, *LR* Laparoscopic Resection, *PHT* Portal Hypertension, *LRFA* Laparoscopic Radiofrequency Ablation, *HCC* Hepatocellular Carcinoma, *BCLC* Barcelona Clinic Liver Cancer, *LLR* Laparoscopic Liver Resection, *DFS* Disease-Free Survival, *MIH* Minimally Invasive Hepatectomy, *PRFA* Percutaneous Radiofrequency Ablation, *E-RFA* Endoscopic Radiofrequency Ablation, *E-pHR* Endoscopic Percutaneous Heat Radiofrequency Ablation, *MIS* Minimally Invasive Surgery, *RFS* Recurrence-Free Survival, *PSM* Propensity Score Matching, *OS* Overall survival, *EVs* Esophageal Varices

### Efficacy outcomes

#### Overall survival

**Fig. 2:**
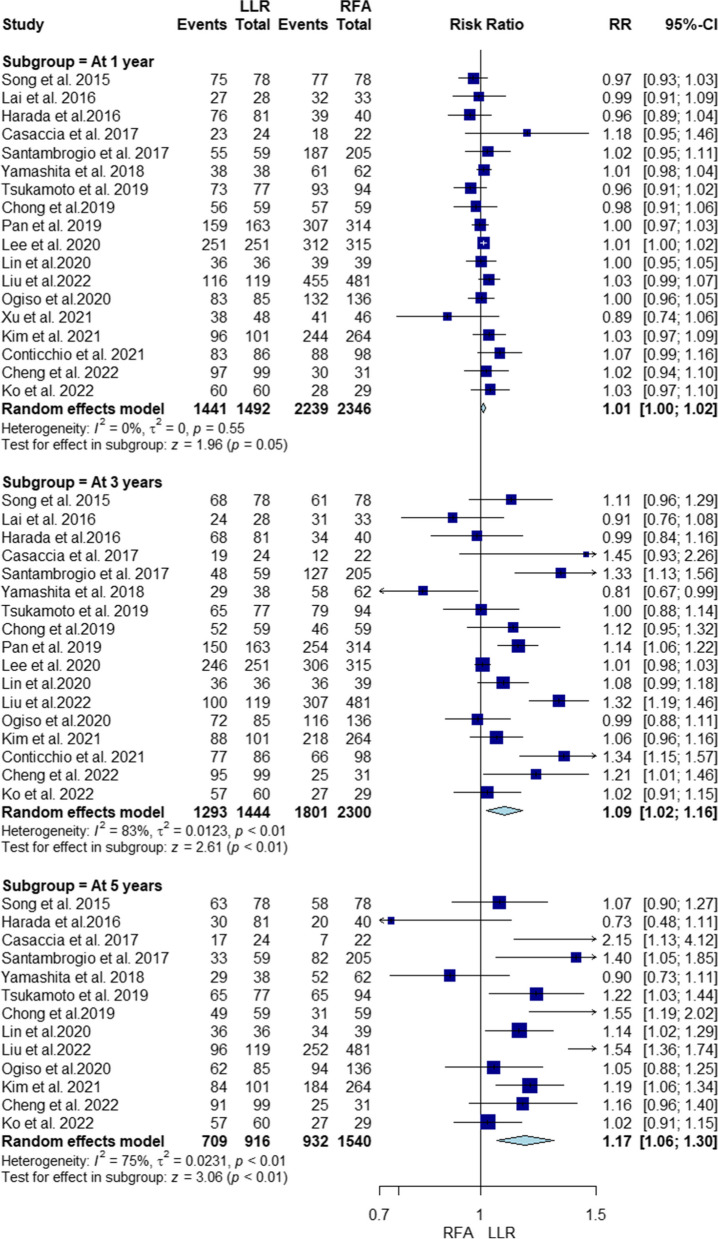
Forest plot Illustrating overall survival

Our pooled analysis showed that the overall survival rate at 1, 3, and 5 years was significantly higher with LLR compared to RFA (RR = 1.01, 95% CI [1, 1.02], *P* = 0.05; RR = 1.09, 95% CI [1.02, 1.16], *P* < 0.01; RR = 1.17, 95% CI [1.06, 1.3], *P* < 0.01, respectively). The pooled studies at 1 year were homogenous (I^2^ = 0%, *P* = 0.55). However, the pooled studies at 3 and 5 years were heterogenous (I^2^ = 83%, *P* < 0.01; I^2^ = 75%, *P* < 0.01, respectively) (Fig. 2). Heterogeneity at 3 and 5 years was not resolved by sensitivity analysis (Supplementary file Figs. S1 and S2, respectively).

Additionally, our subgroup analysis for 1-year overall survival based on RFA type found no significant difference between PRFA or LRFA and LLR (RR = 1.01, 95% CI [1, 1.02], *P* = 0.09; RR = 1.01, 95% CI [0.96, 1.07], *P* = 0.64, respectively), with homogeneity in both subgroups (I2 = 0%, *P* = 0.57; I2 = 46%, *P* = 0.14) (Supplementary file Fig. S3).

For 3-year survival, LLR significantly improved rates compared to PRFA (RR = 1.08, 95% CI [1, 1.16], *P* = 0.05), while no difference was seen between LRFA and LLR (RR = 1.13, 95% CI [0.96, 1.34], *P* = 0.14). Studies were heterogeneous in both subgroups (I2 = 84%, *P* < 0.01; I2 = 74%, *P* < 0.01) (Supplementary file Fig. S4). Heterogeneity in the laparoscopic subgroup resolved by excluding Santambrogio 2017 [33], but not resolved in the percutaneous subgroup (Supplementary file Figs. S5 and S6).

For 5-year survival, LLR significantly outperformed PRFA (RR = 1.15, 95% CI [1.02, 1.31], *P* = 0.03), but no difference was noted between LRFA and LLR (RR = 1.26, 95% CI [0.98, 1.63], *P* = 0.07). Heterogeneity was present in both subgroups (I2 = 77%, *P* < 0.01; I2 = 81%, *P* < 0.01) (Supplementary file Fig. S 7). Heterogeneity in the percutaneous subgroup resolved by excluding Liu 2022 [36] (Supplementary file Fig. S8), and in the laparoscopic subgroup by excluding Ko 2022 [39] (Supplementary file Fig. S9).

Finally, Meta-regression indicated no significant associations between 1-year overall survival and age (*P* = 0.25), total bilirubin level (*P* = 0.49), alpha-fetoprotein level (*P* = 0.2), tumor size within the range of 1.6 to 3.5 cm (*P* = 0.86) (Supplementary file Fig. S10).

#### Overall survival PSM

LLR significantly improved overall survival PSM at 3 years. However, no significant differences were observed between LLR and RFA in overall survival PSM at 1 and 5 years (RR = 1.1, 95% CI [1.03, 1.18], P < 0.01; RR = 1, 95% CI [0.98, 1.02], *P* = 0.99; RR = 1.06, 95% CI [0.86, 1.31], *P* = 0.6, respectively). While studies at 1 and 3 years were homogeneous, those at 5 years exhibited heterogeneity (I2 = 13%, *P* = 0.33; I2 = 28%, *P* = 0.22; I2 = 82%, *P* < 0.01, respectively) (Supplementary file Fig. S11). Heterogeneity at 5 years was not resolved by sensitivity analysis (Supplementary file Fig. S12).

#### Disease-free survival

**Fig. 3:**
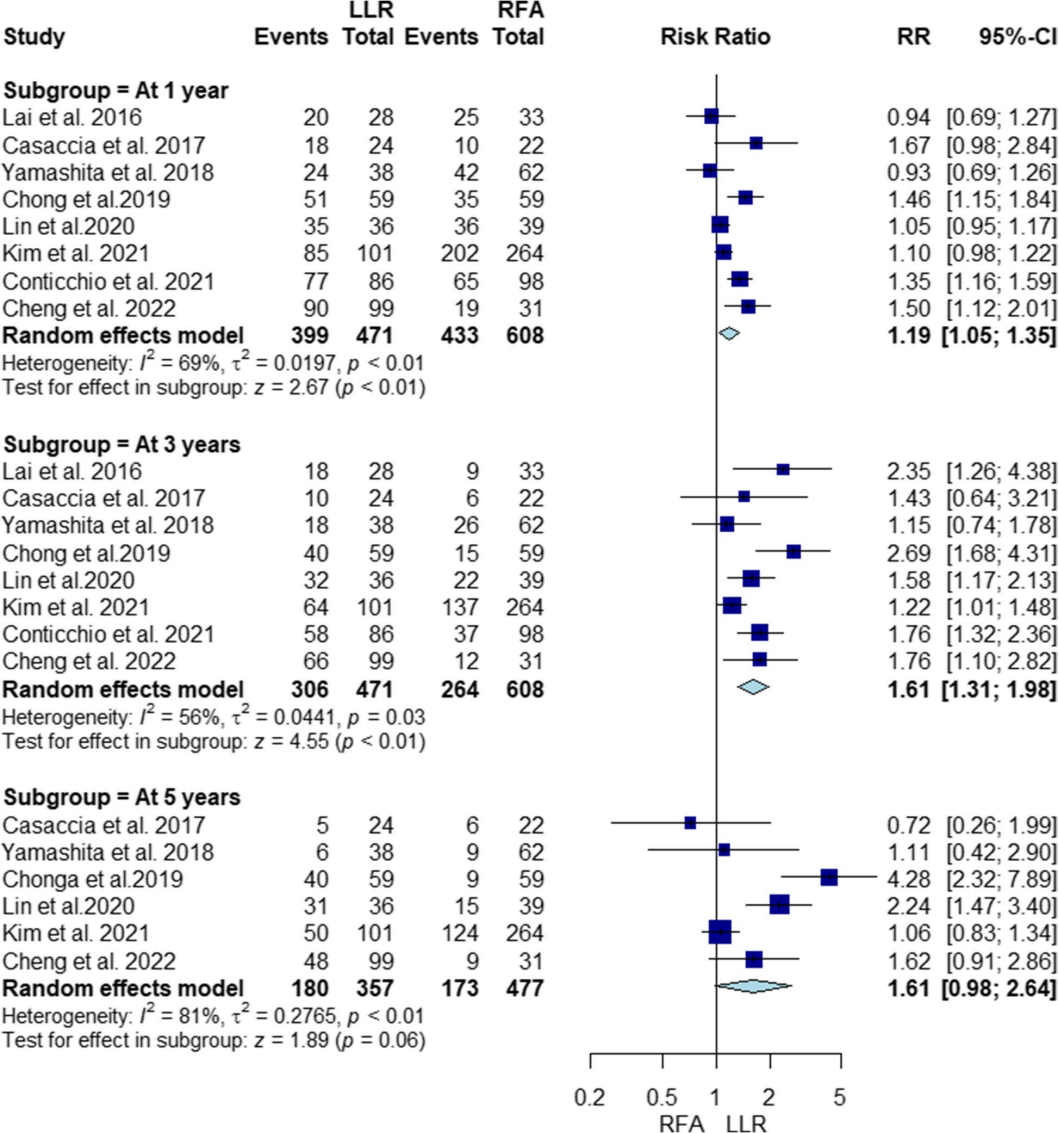
Forest plot Illustrating disease-free survival

Our analysis found higher disease-free survival rates with LLR at 1 and 3 years, but no significant difference between LLR and RFA at 5 years (RR = 1.19, 95% CI [1.05, 1.35], *P* < 0.01; RR = 1.61, 95% CI [1.31, 1.98], *P* < 0.01; RR = 1.61, 95% CI [0.98, 2.64], *P* = 0.06, respectively). Studies at 1, 3, and 5 years were heterogeneous (I2 = 69%, *P* < 0.01; I2 = 56%, *P* = 0.03; I2 = 81%, *P* < 0.01, respectively) (Fig. 3). Heterogeneity at 3 years improved by excluding Kim 2021 [45] (Supplementary file Fig. S13); however, sensitivity analysis did not resolve heterogeneity at 1 and 5 years (Supplementary file Figs. S14 and S15, respectively).

#### Disease-free survival PSM

LLR significantly improved disease-free survival PSM at 1 and 3 years. However, there was no significant difference between LLR and RFA in terms of disease-free survival rate at 5 years (RR = 1.37, 95% CI [1.09, 1.71], *P* < 0.01; RR = 1.99, 95% CI [1.24, 3.2], *P* < 0.01; RR = 2.27, 95% CI [0.78, 6.64], *P* = 0.13, respectively). Studies in all subgroups were heterogeneous (I2 = 74%, *P* = 0.02; I2 = 79%, *P* < 0.01; I2 = 92%, *P* < 0.01, respectively) (Supplementary file Fig. S16). Heterogeneity at 1 year improved by excluding Chong 2019 [41] (Supplementary file Fig. S17); however, sensitivity analysis did not resolve heterogeneity at 3 years (Supplementary file Fig. S18).

#### Recurrence-free survival

**Fig. 4:**
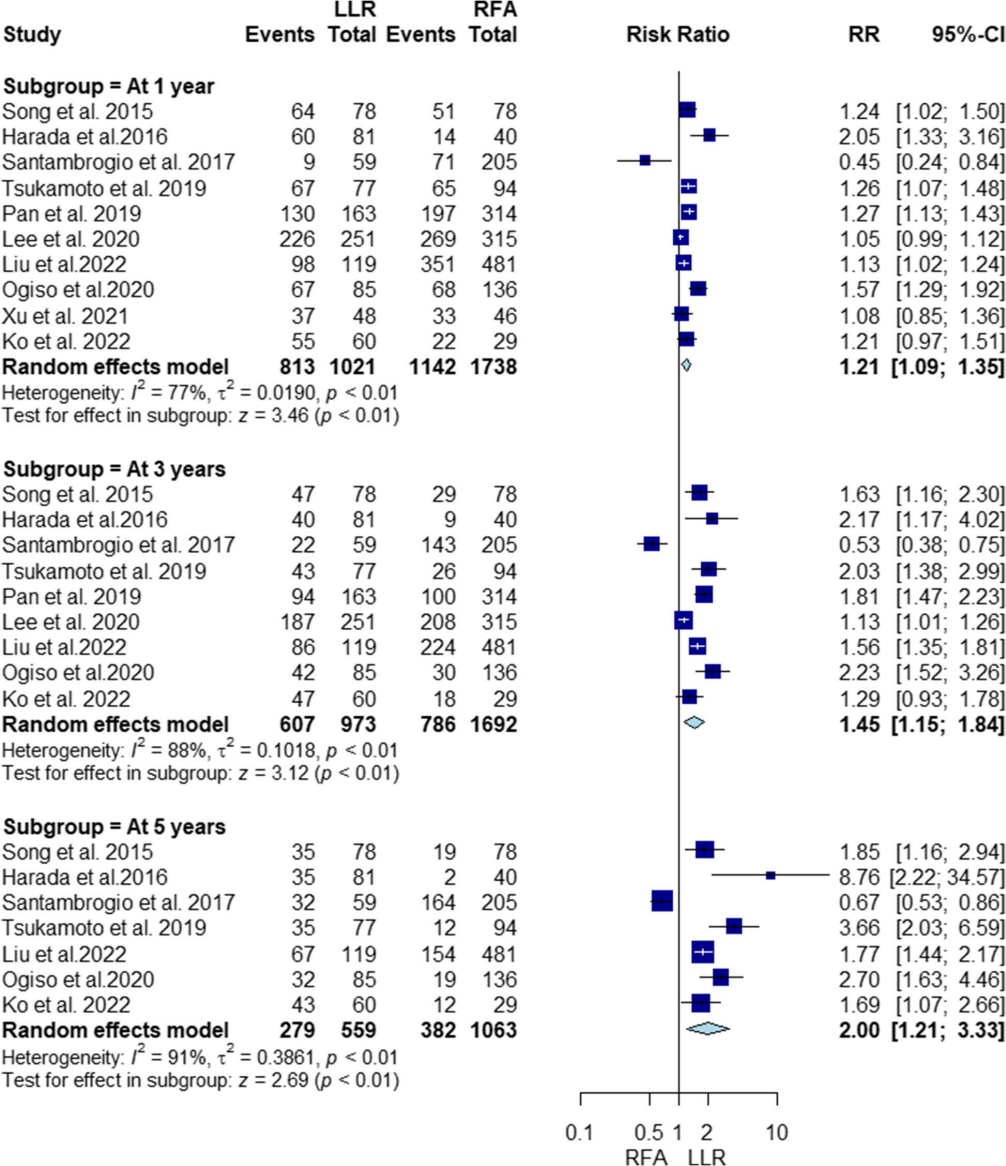
Forest plot Illustrating recurrence-free survival

Our pooled analysis showed that compared to RFA, LLR was associated with higher recurrence-free survival rate at 1, 3, and 5 years (RR = 1.21, 95% CI [1.09, 1.35], *P* < 0.01; RR = 1.45, 95% CI [1.15, 1.84], *P* < 0.01; RR = 2, 95% CI [1.21, 3.33], *P* < 0.01, respectively). The pooled studies at 1, 3, and 5 were heterogenous (I^2^ = 77%, *P* < 0.01; I^2^ = 88%, *P* < 0.01; I^2^ = 91%, *P* < 0.01, respectively) (Fig. 4). Heterogeneity was not resolved by sensitivity analysis (Supplementary file Figs. S19, S20 and S21, respectively).

Our subgroup analysis based on RFA type revealed that LLR was linked to higher recurrence-free survival rates at 1 and 3 years compared to PRFA (RR = 1.24, 95% CI [1.09, 1.41], *P* < 0.01; RR = 1.63, 95% CI [1.29, 2.07], *P* < 0.01, respectively), but no significant difference was observed between LLR and LRFA (RR = 0.99, 95% CI [0.65, 1.51], *P* = 0.97; RR = 1.11, 95% CI [0.52, 2.38], *P* = 0.78, respectively). Heterogeneity was present in both PRFA and LRFA subgroups (I2 = 82%, *P* < 0.01; I2 = 86%, *P* < 0.01; I2 = 85%, *P* < 0.01; I2 = 93%, *P* < 0.01, respectively) (Supplementary file Figs. S22, S23). Heterogeneity in the percutaneous subgroup was partially resolved by omitting Lee 2020 [46] at 1 and 3 years (Supplementary file Figs. S24 and S25 respectively), but not resolved in the laparoscopic subgroup at 1 and 3 years (Supplementary file Figs. S26 and S27 respectively).

Regarding recurrence-free survival at 5 years, LLR was associated with significantly higher rates compared to PRFA (RR = 2.24, 95% CI [1.5, 3.34], *P* < 0.01), while no significant difference was found between LRFA and LLR (RR = 1.57, 95% CI [0.57, 4.33], *P* = 0.39). Both percutaneous and laparoscopic subgroups exhibited heterogeneity (I2 = 64%, *P* = 0.04; I2 = 94%, *P* < 0.01, respectively) (Supplementary file Fig. S28). Heterogeneity in the percutaneous subgroup was partly resolved by omitting Harada 2016 [40], but not resolved in the laparoscopic subgroup (Supplementary file Figs. S29 and S30 respectively).

#### Recurrence-free survival PSM

We found that LLR was associated with higher recurrence-free survival PSM at 1, 3, and 5 years (RR = 1.2, 95% CI [1.04, 1.38], *P* = 0.01; RR = 1, 49% CI [1.1, 2.02], *P* < 0.01; RR = 2.33, 95% CI [1.13, 4.79], *P* = 0.02, respectively). The pooled studies in all subgroups were heterogenous (I^2^ = 71%, *P* < 0.01; I^2^ = 80%, *P* < 0.01; I^2^ = 74%, *P* = 0.02, respectively) (Supplementary file Fig. S31). Heterogeneity at 1 and 5 years was best resolved by omitting Lee 2020 [46] and Harada 2016 [40], respectively (Supplementary file Figs. S32 and S33 respectively) However, heterogeneity at 3 years was not resolved by sensitivity analysis (Supplementary Fig. S34).

#### Local recurrence

**Fig. 5:**
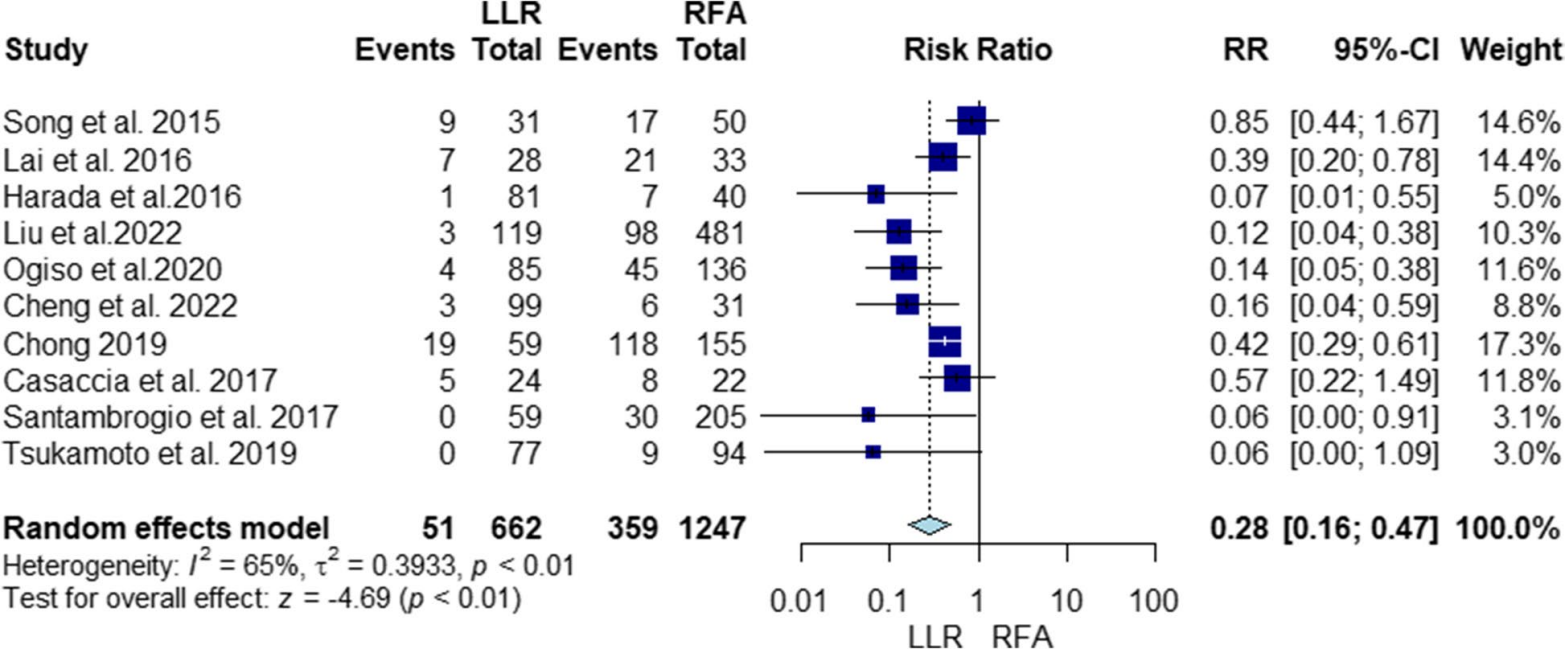
Forest plot Illustrating Local recurrence

**Fig. 6:**
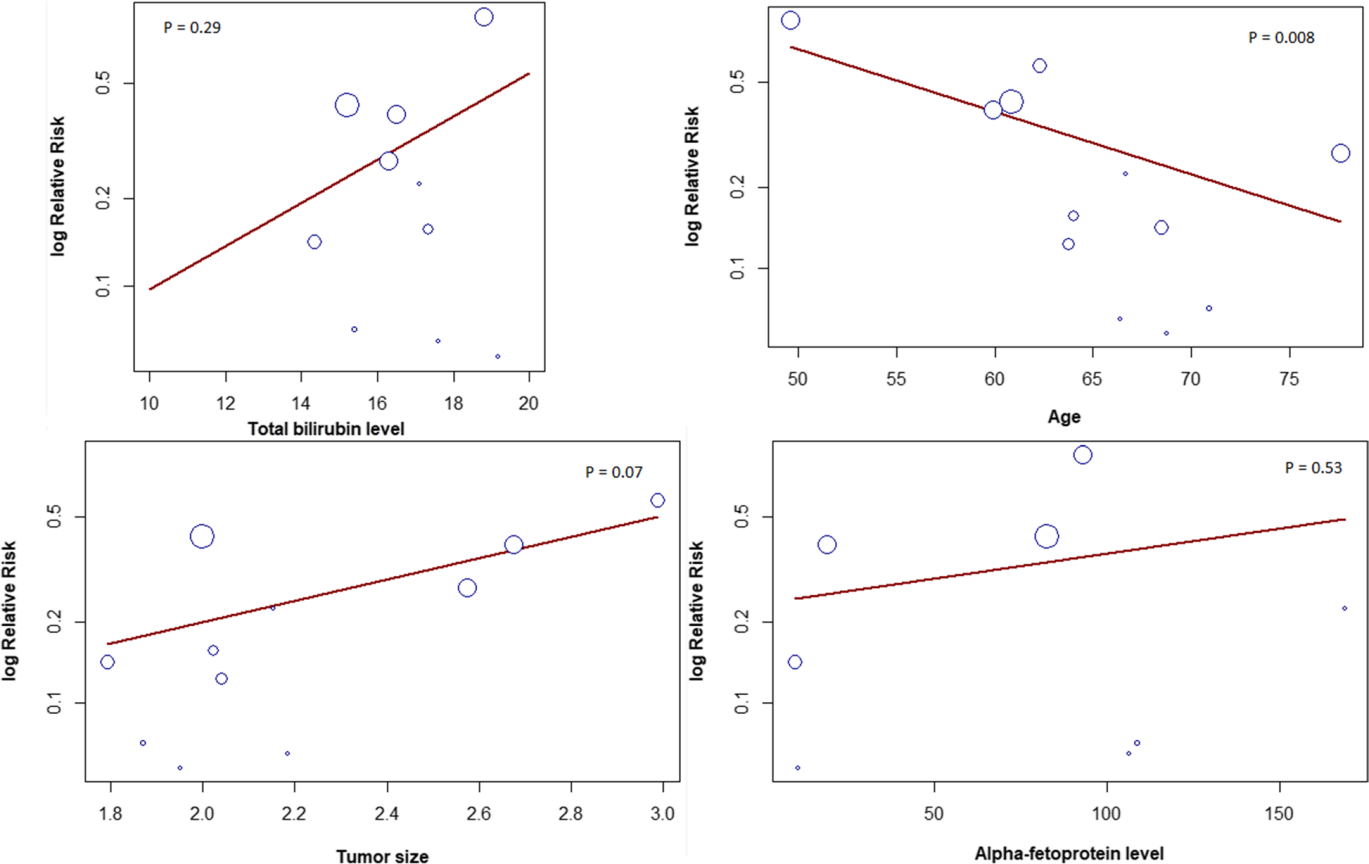
Meta-Regression Analysis of Covariates and local recurrence

The risk for local recurrence was significantly lower with LLR (RR = 0.28, 95% CI [0.16, 0.47], *P* < 0.01). The pooled studies were heterogenous (I^2^ = 65%, *P* < 0.01) (Fig. 5). Heterogeneity was best resolved by omitting Song 2015 [32] (Supplementary file Fig. S35). In addition, our subgroup analysis based on the type of RFA showed that the risk for local recurrence was significantly lower with LLR than with percutaneous RFA; however, there was no significant difference between LLR and laparoscopic RFA (RR = 0.28, 95% CI [0.16, 0.5], *P* < 0.01; RR = 0.16, 95% CI [0.01, 1.84], *P* = 0.65, respectively). The pooled studies were heterogenous in both subgroups (I^2^ = 70%, *P* < 0.01; I^2^ = 74%, *P* = 0.02, respectively) (Supplementary file Fig. S36). Heterogeneity in both subgroups was not resolved by sensitivity analysis (Supplementary file Figs. S37 and S38, respectively). Finally, the results of meta-regression indicated significant association between the risk for local recurrence and the age (*P* = 0.008) (Fig. 6). In contrast, there was no significant association between the risk for local recurrence and the tumor size (*P* = 0.07), alpha-fetoprotein level (*P* = 0.53) and total bilirubin level (*P* = 0.29) (Fig. 6).

#### Intrahepatic recurrence

The risk for intrahepatic recurrence was significantly lower with LLR (RR = 0.7, 95% CI [0.5, 0.97], *P* = 0.03). The pooled studies were heterogenous (I^2^ = 72%, *P* < 0.01) (Supplementary file Fig. S39). Heterogeneity was best resolved by omitting Chong 2019 [41] (Supplementary file Fig. S40).

#### Extrahepatic recurrence

There was no significant difference between LLR and RFA in terms of extrahepatic recurrence (RR = 1.41, 95% CI [0.62, 3.2], *P* = 0.41). The pooled studies were heterogenous (I^2^ = 0%, *P* = 0.83) (Supplementary file Fig. S41).

#### Duration of surgery

The duration of surgery was significantly higher with LLR (SMD = 2.78, 95% CI [1.38, 4. 18], *P* < 0.01). The pooled studies were heterogenous (I^2^ = 98%, *P* < 0.01) (Supplementary file Fig. S42). Heterogeneity was not resolved by sensitivity analysis (Supplementary file Fig. S43).

#### Incidence of blood transfusion during surgery

LLR was associated with higher incidence of blood transfusion compared to RFA (RR = 4.14, 95% CI [1.33, 12.88], *P* = 0.01). The pooled studies were homogenous (I^2^ = 42%, *P* = 0.14) (Supplementary file Fig. S44).

### Safety outcomes

#### All complications

The risk for all complications was significantly higher with LLR (RR = 2.01, 95% CI [1.51, 2.68], *P* < 0.01). The pooled studies were homogenous (I^2^ = 36%, *P* = 0.1) (Supplementary file Fig. S45). Comprehensive details on complications have been incorporated into Table 1.

#### 90-days mortality

The risk for 90-days mortality was significantly lower with LLR (RR = 0.54, 95% CI [0.36, 0. 81], *P* < 0.01). The pooled studies were homogenous (I^2^ = 0%, *P* = 0.9) (Supplementary file Fig. S46).

#### 30-days mortality

The risk for 30-days mortality was significantly higher with LLR (RR = 3.42, 95% CI [1.5, 7. 79], *P* < 0.01). The pooled studies were homogenous (I^2^ = 0%, *P* = 0.39) (Supplementary file Fig. S47).

#### Major complications

The risk for major complications was significantly higher with LLR (RR = 2.02, 95% CI [1.26, 3. 24], *P* < 0.01). The pooled studies were homogenous (I^2^ = 0%, *P* = 0.83) (Supplementary file Fig. S48).

#### Duration of hospital stay

The duration of hospital stay was significantly higher with LLR (SMD = 1.14, 95% CI [0.66, 1. 62], *P* < 0.01). The pooled studies were heterogenous (I^2^ = 92%, *P* < 0.01) (Supplementary file Fig. S49). Heterogeneity was not resolved by sensitivity analysis (Supplementary file Fig. S50).

### Publication bias

The funnel plots for the overall survival at 1, 3, and 5 years were symmetrical. This was confirmed by the insignificant results of Egger’s test (*P* = 0.7; *P* = 0.1; *P* = 0.98, respectively), indicating that there was no publication bias in terms of overall survival at 1, 3, and 5 years. In contrast, visual inspection of the funnel plot for the local recurrence showed asymmetry, which was confirmed by the significant results of Egger’s test (*P* = 0.03) (Supplementary file Fig. S51). Finally, the trim and fill analysis revealed that adding five studies showed that LLR was associated with lower risk for local recurrence (RR = 0.41, 95% CI [0.26; 0.64], *P* < 0.01), which was consistent with our findings (Supplementary Fig. S52).

## Discussion

### Summary of the findings

In our meta-analysis, LLR demonstrated higher overall survival (OS) at 1, 3, and 5 years compared to RFA. Subgroup analysis found no significant OS differences at 1 and 5 years among PRFA, LRFA, and LLR, while LLR exhibited improved 3-year survival over PRFA. Notably, LRFA showed no significant difference from LLR. Meta-regression analysis found no significant associations between 1-year OS and factors such as age, bilirubin, AFP, or tumor size. OS Propensity-score matching indicated a significant improvement at 3 years with LLR, while no differences were observed at 1 and 5 years.

LLR demonstrated enhanced disease-free survival at 1 and 3 years, and recurrence-free survival analysis favored LLR at 1, 3, and 5 years, particularly over PRFA, but no significant difference was found with LRFA.

LLR exhibited significantly lower local recurrence rates compared to RFA, with PRFA showing a notable reduction; however, no significant difference was seen with LRFA. Meta-regression linked this reduction to age. LLR showcased benefits in decreasing intrahepatic recurrence and 90-day mortality; however, it was associated with longer surgery, higher transfusion rates, more complications, and extended hospital stays. We summarized the results of our analysis in Table 2.

**Table 2:** Summary of our analysis

Analysis		RR and 95%CI	*P*-value	Heterogeneity		No. of studies	Conclusion	Figure
				*p*-value	I2			
Overall survival at 1 year		1.01, 95% CI [1, 1.02]	*p* = 0.05	*P* = 0.55	I^2^ = 0%,	18	Higher with LLR compared to RFA	Fig. 2
Type of RFA	PRFA	1.01, 95% CI [1, 1.02	*p* = 0.09	*P* = 0.57	I2 = 0%	11	No significant difference between PRFA and LLR	Supplementary file Fig. S3
	LRFA	1.01, 95% CI [0.96, 1.07]	*P* = 0.64	*P* = 0.14	I2 = 46%	4	No significant difference between LRFA and LLR	Supplementary file Fig. S3
Overall survival at 3 years		1.09, 95% CI [1.02, 1.16]	*P* < 0.01	*P* < 0.01	I^2^ = 83%,	17	Higher with LLR compared to RFA	Fig. 2
Type of RFA	PRFA	1.08, 95% CI [1, 1.16]	*P* = 0.05	*P* < 0.01	I2 = 84%	10	LLR significantly outperformed PRFA	Supplementary file Fig. S4
	LRFA	1.13, 95% CI [0.96, 1.34]	*P* = 0.14	*P* < 0.01	I2 = 74%	4	No significant difference between LRFA and LLR	Supplementary file Fig. S4
Overall survival at 5 years		1.17, 95% CI [1.06, 1.3]	*P* < 0.01	*P* < 0.01	I^2^ = 75%	13	higher with LLR compared to RFA	Fig. 2
Type of RFA	PRFA	1.15, 95% CI [1.02, 1.31]	*P* = 0.03	*P* < 0.01	I2 = 77%	7	LLR significantly outperformed PRFA	Supplementary file Fig. S7
	LRFA	1.26, 95% CI [0.98, 1.63]	*P* = 0.07	*P* < 0.01	I2 = 81%	5	No significant difference between LRFA and LLR	Supplementary file Fig. S7
Overall survival PSM at 1 year		1, 95% CI [0.98, 1.02],	*P* = 0.99	*P* = 0.33	I2 = 13%	7	No significant differences were observed between LLR and RFA	Supplementary file Fig. S11
Overall survival PSM at 3 years		1.1, 95% CI [1.03, 1.18]	*P* < 0.01	*P* = 0.22	I2 = 28%	7	Higher with LLR compared to RFA	Supplementary file Fig. S11
Overall survival PSM at 5 years		1.06, 95% CI [0.86, 1.31]	*P* = 0.6	*P* < 0.01	I2 = 82%	5	No significant differences were observed between LLR and RFA	Supplementary file Fig. S11
Disease-free survival at 1 year		1.19, 95% CI [1.05, 1.35]	*P* < 0.01	*P* < 0.01	I2 = 69%	8	Higher with LLR compared to RFA	Fig. 3
Disease-free survival at 3 years		1.61, 95% CI [1.31, 1.98]	*P* < 0.01	*P* = 0.03	I2 = 56%	8	Higher with LLR compared to RFA	Fig. 3
Disease-free survival at 5 years		1.61, 95% CI [0.98, 2.64]	*P* = 0.06	*P* < 0.01	I2 = 81%	6	No significant differences were observed between LLR and RFA	Fig. 3
Disease-free survival PSM at 1 year		1.37, 95% CI [1.09, 1.71]	*P* < 0.01	*P* = 0.02	I2 = 74%	3	Higher with LLR compared to RFA	Supplementary file Fig. S16
Disease-free survival PSM at 3 years		1.99, 95% CI [1.24, 3.2]	*P* < 0.01	*P* < 0.01	I2 = 79%	3	Higher with LLR compared to RFA	Supplementary file Fig. S16
Disease-free survival PSM at 5 years		2.27, 95% CI [0.78, 6.64]	*P* = 0.13	*P* < 0.01	I2 = 92%	2	No significant differences were observed between LLR and RFA	Supplementary file Fig. S16
Recurrence-free survival at 1 year		1.21, 95% CI [1.09, 1.35]	*P* < 0.01	*P* < 0.01	I^2^ = 77%	10	Higher with LLR compared to RFA	Fig. 4
Type of RFA	PRFA	1.24, 95% CI [1.09, 1.41]	*P* < 0.01	*P* < 0.01	I2 = 82%	7	LLR significantly outperformed PRFA	Supplementary file Fig. S22
	LRFA	0.99, 95% CI [0.65, 1.51]	*P* = 0.97	*P* < 0.01	I2 = 85%	3	no significant difference was observed between LLR and LRFA	Supplementary file Fig. S22
Recurrence-free survival at 3 years		1.45, 95% CI [1.15, 1.84]	*P* < 0.01	*P* < 0.01	I^2^ = 88%	9	Higher with LLR compared to RFA	Fig. 4
Type of RFA	PRFA	1.63, 95% CI [1.29, 2.07]	*P* < 0.01	*P* < 0.01	I2 = 86%	6	LLR significantly outperformed PRFA	Supplementary file Fig. S23
	LRFA	1.11, 95% CI [0.52, 2.38]	*P* = 0.78	*P* < 0.01	I2 = 93%	3	no significant difference was observed between LLR and LRFA	Supplementary file Fig. S23
Recurrence-free survival at 5 years		2, 95% CI [1.21, 3.33]	*P* < 0.01	*P* < 0.01	I^2^ = 91%	7	Higher with LLR compared to RFA	Fig. 4
Type of RFA	PRFA	2.24, 95% CI [1.5, 3.34]	*P* < 0.01	*P* = 0.04	I2 = 64%	4	LLR significantly outperformed PRFA	Supplementary file Fig. S28
	LRFA	1.57, 95% CI [0.57, 4.33]	*P* = 0.39	P < 0.01	I2 = 94%	3	no significant difference was observed between LLR and LRFA	Supplementary file Fig. S28
Recurrence-free survival PSM at 1 year		1.2, 95% CI [1.04, 1.38]	*P* = 0.01	*P* < 0.01	I^2^ = 71%	5	Higher with LLR compared to RFA	Supplementary file Fig. S31
Recurrence-free survival PSM at 3 years		1, 49% CI [1.1, 2.02]	*P* < 0.01	*P* < 0.01	I^2^ = 80%	5	Higher with LLR compared to RFA	Supplementary file Fig. S31
Recurrence-free survival PSM at 5 years		2.33, 95% CI [1.13, 4.79]	*P* = 0.02	*P* = 0.02	I^2^ = 74%	3	Higher with LLR compared to RFA	Supplementary file Fig. S31
Local recurrence		0.28, 95% CI [0.16, 0.47]	*P* < 0.01	*P* < 0.01	I^2^ = 65%	10	Significantly lower with LLR	Fig. 5
Type of RFA	PRFA	0.28, 95% CI [0.16, 0.5]	*P* < 0.01	*P* < 0.01	I^2^ = 70%	7	Significantly lower with LLR	Supplementary file Fig. S36
	LRFA	0.16, 95% CI [0.01, 1.84]	*P* = 0.65	*P* = 0.02	I^2^ = 74%	3	no significant difference was observed between LLR and LRFA	Supplementary file Fig. S36
Intrahepatic recurrence		0.7, 95% CI [0.5, 0.97]	*P* = 0.03	*P* < 0.01	I^2^ = 72%	8	Significantly lower with LLR	Supplementary file Fig. S39
Extrahepatic recurrence		1.41, 95% CI [0.62, 3.2]	*P* = 0.41	*P* = 0.83	I^2^ = 0%	4	no significant difference between LLR and RFA	Supplementary file Fig. S41
Duration of surgery		SMD = 2.78, 95% CI [1.38, 4. 18]	*P* < 0.01	*P* < 0.01	I^2^ = 98%	8	Significantly higher with LLR	Supplementary file Fig. S42
Incidence of blood transfusion during surgery		4.14, 95% CI [1.33, 12.88]	*P* = 0.01	*P* = 0.14	I^2^ = 42%	5	Significantly higher with LLR	Supplementary file Fig. S44
All complications		2.01, 95% CI [1.51, 2.68]	*P* < 0.01	*P* = 0.1	I^2^ = 36%	13	Significantly higher with LLR	Supplementary file Fig. S45
90-days mortality		0.54, 95% CI [0.36, 0. 81]	*P* < 0.01	*P* = *0.9*	*I2* = *0%*	4	Significantly lower with LLR	Supplementary file Fig. S46
30-days mortality		3.42, 95% CI [1.5, 7. 79]	*P* < 0.01	*P* = *0.39*	*I2* = *0%*	7	Significantly higher with LLR	Supplementary file Fig. S47
Major complications		2.02, 95% CI [1.26, 3. 24]	*P* < 0.01	*P* = 0.83	I^2^ = 0%	9	Significantly higher with LLR	Supplementary file Fig. S48
Duration of hospital stay		SMD = 1.14, 95% CI [0.66, 1. 62]	*P* < 0.01	*P* < 0.01	I^2^ = 92%	10	Significantly higher with LLR	Supplementary file Fig. S49

### Explanation of the findings

Open hepatectomy (OH) is a well-established method for treating HCCs, but its drawbacks include large incisions, extensive resection, and significant blood loss causing trauma. OH, suits patients with normal liver function; however, it is not suitable for patients with severe cirrhosis. A recent analysis showed that laparoscopic liver resection (LLR) was associated with lower postoperative complications, such as ascites and liver failure than OH. Therefore, LLR emerged as a minimally invasive alternative for OH, particularly in patients with severe cirrhosis [47, 48].

However, not all cases are suitable for LLR because LLR is primarily indicated for easily reachable lesions and tumors in the outer part of anterolateral liver segments (segments 2, 3, 5, and 6). Lesions in the posterior or upper liver regions (segments 1, 7, and 8, and the upper part of segment 4) represent technical challenges due to bleeding control and limited visibility difficulties [49, 50]. LLR is particularly considered the preferred option for small HCC cases, even in cirrhotic patients, when feasible, as its effectiveness matches that of open surgery in achieving a cure [51].

Radiofrequency ablation (RFA) is a widely used minimally invasive approach for treating HCCs. Various randomized controlled trials (RCTs) and meta-analyses have compared RFA with OH [52, 53]. These studies have consistently demonstrated that RFA is effective for early-stage HCCs, offering comparable prognostic outcomes and a lower complication rate than OH. In recent years, this has led to an increasing focus among surgeons on comparing these minimally invasive methods for the curative treatment of HCCs.

Advancements in artificial hydrothorax, imaging-guided localization, and probes have considerably expanded the indications of RFA. RFA procedures are performed under conscious sedation. Furthermore, most patients undergoing RFA treatment experience brief hospital stays of 2 to 3 days; in some cases, they can even be discharged on the same day, eliminating the need for prolonged hospitalization [54]. As a result, it is evident that RFA treatment is associated with reduced postoperative complications, shorter surgical durations, and minimized hospitalization periods. It’s a viable supplemental therapy for cirrhotic livers without significant damage.

However, local recurrence at the RFA treatment site is a common limitation. Rhim et al. noted this due to limited ablation volume, technical difficulties for certain tumors based on location, and the heat sink effect caused by nearby large vessels. [55]. Therefore, our observations of the higher local recurrence rates may be attributed to the incomplete ablation of the primary HCC tumor, the heat sink impact, or venous invasion in the adjacent liver. On the other hand, LLR provides a broader safety margin during treatment and often involves completely removing segments containing tumors. This thorough approach may contribute to lower recurrence rates in HCC patients with LLR [56].

In our subgroup analyses, we found that LLR had better outcomes for OS, RFS, and local recurrence rates compared to PRFA. However, regarding 1 to 5 years of OS, RFS, and local recurrence rates, LRFA and LLR had similar effects. This may be attributed to the ability of laparoscopic techniques to detect microscopic tumor foci. In addition, laparoscopic approaches allow precise electrode placement, especially in difficult tumor locations, through comprehensive exploration and intraoperative ultrasound. [57] Laparoscopic RFA's superiority over the percutaneous approach, especially in complex cases or severe liver disease, broadens the scope of RFA treatments, effectively expanding their applications [58].

The findings from the meta-regression analysis demonstrate that certain factors significantly impact the local recurrence in early-stage HCC. Specifically, the analysis reveals a noteworthy correlation between the age of the patient and the incidence of recurrence.

It is interesting to note that there is an inverse correlation between age and recurrence risk, which may seem counterintuitive since one might expect older patients to have a higher risk due to compromised immune function and overall health. However, this observation is consistent with earlier studies on older breast cancer patients conducted by Anna Z. de Boer et al. in 2020, which found that individuals aged 75–79 were more likely to experience distant recurrence but not locoregional recurrence risk [59]. Similarly, research by R. A. M. Damhuis et al. in 1997 demonstrated that older age was associated with reduced local recurrence rates in rectal cancer across three different age groups (15–64, 65–74, and 75 and over). [60] Thus, advancing age may decrease local recurrence rates but potentially increase the likelihood of distant recurrence in the context of HCC.

However, it is essential to note that the variability in study designs and patient populations across the included studies limits our findings. Further research is needed to explore the molecular mechanisms and interactions with other unexplored factors.

Also, our meta-regression analysis found no significant link between tumor size and overall survival or local recurrence in HCC patients, challenging the prior consensus associating larger tumor size with worse outcomes [61, 62].

Interestingly, Anli Yang et al.'s [63] research has also found that for patients without vascular invasion, tumor size matters notably for overall survival in the radiofrequency ablation group, but this association is not observed in either the liver resection or transplantation group. Conversely, for patients with vascular invasion, tumor size affects survival in the liver resection and transplantation group. These findings suggest two possibilities: tumor size may not be as crucial a prognostic factor in HCC as believed, with factors like tumor stage, vascular invasion, and liver function playing more significant roles. Additionally, the relationship between tumor size and HCC survival may be more complex, influenced by age, gender, or underlying liver disease. So, the clinicians should be cautious about relying solely on tumor size for treatment decisions and consider multiple factors for more informed choices.

Given these uncertainties, further research is needed to better understand the tumor size and survival relationship in HCC.

In comparison to the previous meta-analyses conducted by Mou‐Bo Si in 2019 [16], Xiaocheng Li in 2019 [20], Shan Jin in 2020 [17], and Zhijun Li in 2021 [19], our current study provides a thorough and up-to-date assessment of various liver resection techniques, with a particular emphasis on the benefits associated with LLR and RFA approaches.

### Agreements and disagreements with previous studies

Our analysis incorporates 19 studies and a substantial pooled sample size of 3756 patients, as presented in Table 3. Prior studies had differing numbers of included studies, ranging from 6 to 19, and sample sizes ranging from 597 to 2038.

**Table 3:** Comparison of our meta-analysis with another published meta-analysis

	**Mou‐Bo Si 2019 **[16]	**Xiaocheng Li 2019 **[20]	**Shan Jin 2020 **[17]	**Zhijun Li 2021 **[19]	**Our study**
Total studies included in MA	6	10	7	19	19
Total sample size	597	1570	615	2038	3756
Language of included studies	English	English	English	3 in English,16 in Chinese	English
Number of outcomes analyzed	6	4	5	9	16
Subgroup analysis based on	Tumor size and RFA approaches	Tumor sizes	NR	Tumor sizes, RFA approaches and study areas in China	RFA approaches
Meta regression covariates	NR	NR	NR	NR	age, tumor size, total bilirubin, and alpha-fetoprotein
OS, DFS, RFS measured at	At 1 year, and 3 years	At 1year,3 years, and 5 years	NR	At 1 year, and 3 years	At 1year,3 years, and 5 years
Overall survival	At 1year no statistical differences At 3 years favors MIS group	Favors LH at 1year,3 years, and 5 years	NR	At 1year no statistical differences At 3 years favors LLR group	Favors LLR group at 1year,3 years, and 5 years
Overall survival PSM	NR	NR	NR	NR	At 3 years Favors LLR group, At 1and 5 years no statistical differences
Disease‐free survival	At 1 year and 3 years favors MIS group	At 1 year and 3 years favors LH At 5 years no statistical differences	NR	At 1 year and 3 years favors LLR group	At 1 year and 3 years favors LLR group, at 5 years no statistical differences
Disease‐free survival PSM	NR	NR	NR	NR	At 1 year and 3 years favors LLR group, at 5 years no statistical differences
Recurrence-free survival	NR	NR	NR	NR	Favors LLR group
Recurrence-free survival PSM	NR	NR	NR	NR	Favors LLR group
Overall response rate	NR	NR	NR	Favors RFA group	NR
Local recurrence	Higher with RFA group	Higher with RFA group	Higher with RFA group	Higher with RFA group	Higher with RFA group
Intrahepatic recurrence	NR	NR	NR	NR	Higher with RFA group
Extrahepatic recurrence	NR	NR	NR	NR	no statistical differences
Postoperative liver function index	NR	NR	NR	RFA group had lower AST and higher ALB levels, with no significant differences in ALT, TBIL, and AFP levels	NR
Duration of surgery	Lower with RFA group	NR	Lower with RFA group	Lower with RFA group	Lower with RFA group
Duration of hospital stay	Lower with RFA group	NR	Lower with RFA group	NR	Lower with RFA group
Incidence of blood transfusion during surgery	NR	NR	Lower with RFA group	Lower with RFA group	Lower with RFA group
Estimated bleeding volume during surgery	NR	NR	Lower with RFA group	Lower with RFA group	NR
All complications	Higher with MIS	Higher with LH group	NR	Higher with LLR group	Higher with LLR group
30-days mortality	NR	NR	NR	NR	lower with LLR group
90-days mortality	NR	NR	NR	NR	Higher with LLR group
Major complications	NR	NR	NR	NR	Higher with LLR group

*Abbreviations*: *MA* Meta-analysis, *RFA* Radiofrequency ablation, *OS* Overall survival, *DFS* Disease free survival, *RFS* Recurrence free survival, *MIS* Minimally invasive liver surgery, *LH* Laparoscopic hepatectomy, *LLR* Laparoscopic liver resection, *PSM* Propensity-score matching, *AST* Aspartate Aminotransferase, *ALB* Albumin, *ALT* Alanine Aminotransferase, *TBIL* Total Bilirubin, *AFP* Alpha-Fetoprotein, *NR* Not reported

After conducting a thorough and detailed analysis, we have discovered significant differences and similarities in the results of various meta-analyses. In the context of overall survival, our findings closely align with those of Xiaocheng Li et al. [20]. However, when examining the research conducted by Mou‐Bo Si et al. [16] and Zhijun Li et al. [19], their results demonstrate that there were no statistically significant differences observed at the 1 year, whereas the outcomes favored the LLR group at 3 years.

On the other hand, all the meta-analyses indicate that the LLR group has a better disease-free survival rate at one and three years. However, at five years, our study and Xiaocheng Li's et al. [20] highlight a lack of statistical differences.

Across all meta-analyses [16, 17, 19, 20], the RFA group consistently shows higher local recurrence rates and shorter duration of both surgery and hospital stay compared to the LLR group. Additionally, complications are uniformly more prevalent in the LLR group according to all analyses.

### Strength points and limitations

To date, our study is the most comprehensive meta-analysis comparing LLR versus RFA in patients with early-stage HCC. We included 19 observational studies, compromising 3756 patients. We covered a five-year follow-up period, analyzing OS, DFS, and RFS using Propensity Score Matching while examining Intrahepatic and Extrahepatic recurrence. In addition, we comprehensively evaluated safety measures in terms of all complications, 30-day and 90-day mortality, and major complications. Moreover, our study is the first meta-analysis in this topic to conduct subgroup analysis based on RFA type, including four laparoscopic RFA studies, which is a significant improvement compared to previous meta-analyses that only featured one study. Finally, our study is the first to perform meta-regression analysis to explore the association between overall survival and local recurrence with multiple covariates such as age, tumor size, total bilirubin, and alpha-fetoprotein.

In addressing the limitations of our analysis, it's crucial to emphasize that our study exclusively incorporated English-language studies. It's also essential to acknowledge that most of the studies we examined were retrospective, potentially introducing an increased risk of bias, particularly concerning the selection of patients. The varying availability of resources and diverse levels of expertise among medical practitioners might have significantly influenced treatment choices, constraining our findings' broader applicability. Moreover, we observed heterogeneity across different outcomes, and indications of publication bias emerged in multiple studies. Our analysis did not compare outcomes such as quality of life, liver functions after treatment, and overall response rate as these data were not reported in our included studies. Furthermore, our ability to perform a subgroup analysis based on portal hypertension, cirrhosis, etiology of the underlying disease, or tumor location was hindered by inherent constraints.

### Implications of our findings in practice

Based on our study, LLR provides better long-term survival outcomes at 1, 3, and 5 years compared to RFA, making it the preferred option. However, subgroup analysis indicates that LRFA yields similar survival rates to LLR at these time intervals, providing a less invasive alternative. It is important to consider individual patient characteristics and preferences when making treatment decisions. LLR has advantages in terms of disease-free and recurrence-free survival, especially over PRFA. Age has been identified as a factor in reducing local recurrence rates.

Additionally, our research indicates that tumor size may not be as critical a prognostic factor in HCC as previously thought. This information can aid clinicians in making treatment decisions. For instance, clinicians may be less inclined to exclude patients from surgery solely based on tumor size.

Nevertheless, clinicians must balance these benefits against LLR's longer surgery times, higher transfusion rates, complications, and extended hospital stays. Additionally, the study highlights the potential of laparoscopic RFA techniques, as no significant differences were found between LLR and LRFA in several key outcomes, suggesting future research in this area.

### Recommendations

To improve HCC management, t is recommended to conduct larger, long-term comparative studies and prioritize well-designed randomized controlled trials. These efforts would validate current findings, assess treatment long-term effects, and provide robust evidence. Additionally, considering both survival outcomes and patients' quality of life is crucial, along with evaluating cost-effectiveness for informed healthcare decision-making. It is crucial to explore the impact of evolving technologies on outcomes, especially within laparoscopic radiofrequency ablation techniques. Incorporating patient-reported outcomes and satisfaction assessments can provide valuable insights into treatment preferences.

Furthermore, additional research is needed to comprehensively understand the correlation between tumor size and HCC survival rates. Additionally, exploring the impact of age on local recurrence, as well as both intrahepatic and extrahepatic recurrence, and to identify other covariates influencing overall survival and local recurrence. By conducting more research, we can better understand HCC management and improve patient outcomes.

## Conclusion

In this meta-analysis, LLR yielded better oncological outcomes than RFA for patients with early and small HCC. LLR exhibited superior 5-year overall survival and lower recurrence rates, although it was associated with higher complication rates than RFA. The study also highlighted the potential of enhancing outcomes via laparoscopic RFA techniques, as no significant differences were found between LLR and LRFA in terms of overall survival, recurrence-free survival, and local recurrence. However, it is essential to emphasize that further well-designed prospective studies of high quality are necessary to validate and substantiate the conclusions drawn from this meta-analysis.

### Supplementary Information

**Additional file 1:** **Supplementary figure S1.** Sensitivity analysis of overall survival at 3 years. **Supplementary figure S2.** Sensitivity analysis of overall survival at 5 years. **Supplementary file figure S3.** Forrest plot illustrating subgroup analysis for 1-year overall survival based on RFA type. **Supplementary file figure S4.** Forrest plot illustrating Subgroup analysis for 3-years overall survival based on RFA type. **Supplementary file figure S5.** Sensitivity analysis of laparoscopic subgroup overall survival at 3 years. **Supplementary file figure S6.** Sensitivity analysis of percutaneous subgroup overall survival at 3 years. **Supplementary file figure S7. **Forrest plot illustrating Subgroup analysis for 5-years overall survival based on RFA type. **Supplementary file figure S8.** Sensitivity analysis of percutaneous subgroup overall survival at 5 years. **Supplementary file figure S9. **Sensitivity analysis of laparoscopic subgroup overall survival at 5 years. **Supplementary file figure S10. **Meta-Regression Analysis of Covariates and 1-Year overall Survival. **Supplementary file figure S11. **Forrest plot illustrating overall survival PSM. **Supplementary file figure S12. **Sensitivity analysis of overall survival PSM at 5 years. **Supplementary file Figure S13**. Sensitivity analysis of disease-free survival at 3 years. **Supplementary file Figure S14.** Sensitivity analysis of disease-free survival at 1 year. **Supplementary file Figure S15. **Sensitivity analysis of disease-free survival at 5 years. **Supplementary file Figure S16.** Forrest plot illustrating disease-free survival PSM. **Supplementary file Figure S17. **Sensitivity analysis of disease-free survival PSM at 1 year. **Supplementary file Figure S18.** Sensitivity analysis of disease-free survival PSM at 3 years. **Supplementary file Figure S19.** Sensitivity analysis of recurrence-free survival at 1 year. **Supplementary file Figure S20.** Sensitivity analysis of recurrence-free survival at 3 years. **Supplementary file Figure S21.** Sensitivity analysis of recurrence-free survival at 5 years. **Supplementary file Figure S22.** Forrest plot illustrating subgroup analysis for 1-year recurrence-free survival based on RFA type. **Supplementary file Figure S23.** Forrest plot illustrating subgroup analysis for 3-years recurrence-free survival based on RFA type. **Supplementary file Figure S24.** Sensitivity analysis of percutaneous subgroup recurrence free survival at 1 year. **Supplementary file Figure S25.** Sensitivity analysis of percutaneous subgroup recurrence free survival at 3 years. **Supplementary file Figure S26.** Sensitivity analysis of laparoscopic subgroup recurrence free survival at 1 year. **Supplementary file Figure S27.** Sensitivity analysis of laparoscopic subgroup recurrence free survival at 3 years. **Supplementary file Figure S28.** Forrest plot illustrating Subgroup analysis for 5-years Recurrence-free survival based on RFA type. **Supplementary file Figure S29.** Sensitivity analysis of percutaneous subgroup recurrence free survival at 5 years. **Supplementary file Figure S30.** Sensitivity analysis of laparoscopic subgroup recurrence free survival at 5 years. **Supplementary file Figure S31.** Forrest plot illustrating recurrence-free survival PSM. **Supplementary file Figure S32.** Sensitivity analysis of recurrence-free survival PSM at 1 year. **Supplementary file Figure S33.** Sensitivity analysis of recurrence-free survival PSM at 5 years. **Supplementary file Figure S34.** Sensitivity analysis of recurrence-free survival PSM at 3 years. **Supplementary file Figure S35.** Sensitivity analysis of local recurrence. **Supplementary file Figure S36.** Forrest plot illustrating Subgroup analysis for local recurrence based on RFA type. **Supplementary file Figure S37.** Sensitivity analysis of percutaneous subgroup local recurrence. **Supplementary file Figure S38.** Sensitivity analysis of laparoscopic subgroup local recurrence. **Supplementary file Figure S39.** Forrest plot illustrating intrahepatic recurrence. **Supplementary file Figure S40.** Sensitivity analysis of intrahepatic recurrence. **Supplementary file Figure S41.** Forrest plot illustrating extrahepatic recurrence. **Supplementary file Figure S42.** Forrest plot illustrating duration of surgery. **Supplementary file Figure S43.** Sensitivity analysis of duration of surgery. **Supplementary file Figure S44.** Forrest plot illustrating incidence of blood transfusion during surgery. **Supplementary file Figure S45.** Forrest plot illustrating all complications. **Supplementary file Figure S46.** Forrest plot illustrating 90-days mortality. **Supplementary file Figure S47.** Forrest plot illustrating 30-days mortality. **Supplementary file Figure S48.** Forrest plot illustrating major complications. **Supplementary file Figure S49.** Forrest plot illustrating duration of hospital stay. **Supplementary file Figure S50.** Sensitivity analysis of duration of hospital stay. **Supplementary file Figure S51.** Funnel plot for the local recurrence. **Supplementary file Figure S52.** Funnel plot (trim and fill method) for the local recurrence.

**Additional file 2:** **Supplementary Table 1.** Baseline characteristics of enrolled patients in each included study.

## Data Availability

All data generated or analyzed during this study are included in this published article or in the data repositories listed in References.

## Abbreviations

AFP: Alpha-Fetoprotein
BCLC: Barcelona Clinic Liver Cancer
CI: Confidence Interval
DFS: Disease-Free Survival
HCC: Hepatocellular Carcinoma
HBV: Hepatitis B Virus
HCV: Hepatitis C Virus
LLR: Laparoscopic Liver Resection
LRFA: Laparoscopic Radiofrequency Ablation
NOS: Newcastle-Ottawa Scale
OH: Open Hepatectomy
OS: Overall Survival
PRFA: Percutaneous Radiofrequency Ablation
PSM: Propensity-Score Matching
PRISMA: Preferred Reporting Items for Systematic Reviews and Meta-Analyses
RFA: Radiofrequency Ablation
RR: Risk Ratio
RFS: Recurrence-Free Survival
