# Supplementary material for: Efficacy and safety of laparoscopic liver resection versus radiofrequency ablation in patients with early and small hepatocellular carcinoma: an updated meta-analysis and meta-regression of observational studies
Source: World J Surg Oncol. 2024 Feb 7;22:47. doi: 10.1186/s12957-023-03292-3 (PMC10848480; doi:10.1186/s12957-023-03292-3)
Supplement: Supplementary file 1 — Additional file 1: Supplementary figure S1. Sensitivity analysis of overall survival at 3 years. Supplementary figure S2. Sensitivity analysis of overall survival at 5 years. Supplementary file figure S3. Forrest plot illustrating subgroup analysis for 1-year overall survival based on RFA type. Supplementary file figure S4. Forrest plot illustrating Subgroup analysis for 3-years overall survival based on RFA type. Supplementary file figure S5. Sensitivity analysis of laparoscopic subgroup overall survival at 3 years. Supplementary file figure S6. Sensitivity analysis of percutaneous subgroup overall survival at 3 years. Supplementary file figure S7. Forrest plot illustrating Subgroup analysis for 5-years overall survival based on RFA type. Supplementary file figure S8. Sensitivity analysis of percutaneous subgroup overall survival at 5 years. Supplementary file figure S9. Sensitivity analysis of laparoscopic subgroup overall survival at 5 years. Supplementary file figure S10. Meta-Regression Analysis of Covariates and 1-Year overall Survival. Supplementary file figure S11. Forrest plot illustrating overall survival PSM. Supplementary file figure S12. Sensitivity analysis of overall survival PSM at 5 years. Supplementary file Figure S13. Sensitivity analysis of disease-free survival at 3 years. Supplementary file Figure S14. Sensitivity analysis of disease-free survival at 1 year. Supplementary file Figure S15. Sensitivity analysis of disease-free survival at 5 years. Supplementary file Figure S16. Forrest plot illustrating disease-free survival PSM. Supplementary file Figure S17. Sensitivity analysis of disease-free survival PSM at 1 year. Supplementary file Figure S18. Sensitivity analysis of disease-free survival PSM at 3 years. Supplementary file Figure S19. Sensitivity analysis of recurrence-free survival at 1 year. Supplementary file Figure S20. Sensitivity analysis of recurrence-free survival at 3 years. Supplementary file Figure S21. Sensitivity analysis of r [file 12957_2023_3292_MOESM1_ESM.docx]

**Efficacy and safety of laparoscopic liver resection versus radiofrequency ablation in patients with early and small hepatocellular carcinoma: an updated meta-analysis and meta-regression of observational studies**

**Mahmoud Shaban Abdelgalil^1^*^,^ Basma Ehab Amer^2^, Noha Yasen^3,9^**, **Mohamed El-Samahy ^4^**, **Ahmed K. Awad ^5^, Bahaa Elfakharany ^6^,** **Omar Saeed ^7^, Mohamed Abd-ElGawad^8^**

1. *Faculty of Medicine, Ain-shams University, Cairo, Egypt.*

[*29908068800596@med.asu.edu.eg*](mailto:29908068800596@med.asu.edu.eg)

ORCID: 0000-0002-7325-0129

1. *Faculty of Medicine, Benha University, Benha, Egypt.*

[*basma.ehab15@gmail.com*](mailto:basma.ehab15@gmail.com)

*ORCID: 0000-0001-7787-5508*

1. *Faculty of Applied Medical Sciences, Misr university for science and technology, Cairo, Egypt.*

[*nohayasen1724@gmail.com*](mailto:nohayasen1724@gmail.com)

1. *Faculty of Medicine, Zagazig University, Zagazig, Egypt.*

[sama7y1@gmail.com](mailto:sama7y1@gmail.com)

ORCID*:* 0000-0002-3517-0822

1. *Faculty of Medicine, Ain-shams University, Cairo, Egypt.*

[ahmedkawad@gmail.com](mailto:ahmedkawad@gmail.com)

1. *Faculty of Applied Medical Sciences, Jerash University, Jerash, Jordan.*

[*bahaaelfkharany@gmail.com*](mailto:bahaaelfkharany@gmail.com)

ORCID: 0009-0004-3046-847X

1. *Faculty of Medicine, Ain-shams University, Cairo, Egypt.*

[30007312101431@med.asu.edu.eg](mailto:30007312101431@med.asu.edu.eg)

ORCID: 0000-0001-8333-1742

1. *Faculty of Medicine, Fayoum University, Fayoum, Egypt.*

[mm2953@fayoum.edu.eg](mailto:mm2953@fayoum.edu.eg)

1. *Medical Research Group of Egypt, Negida Academy, Arlington, MA, United States.*

**Corresponding author*:**

Faculty of Medicine, Ain-shams University, Cairo, Egypt.

Postal address; 359 Abd Allah Nadim Street, Cairo, Egypt.

Email: 29908068800596@med.asu.edu.eg

ORCID: 0000-0002-7325-0129

Phone: :(+20)1154257518

**Search strategy: -**

**PubMed**;

1-(Liver neoplasm* OR Hepatic Neoplasm* OR Hepatocellular Cancer* OR Liver Cancer* OR Hepatic Cancer* OR Hepatocellular Carcinoma* OR HCC OR Liver Cancer OR Liver cell Carcinoma* OR Hepatoma* OR hepatic malignanc*)

2- (Laparoscopic OR Laparoscop* OR Celioscop* OR Peritoneoscop* OR Laparoscopic Surgery)

3- (Hepatectomy AND Hepatectomies)

4- (Radiofrequency OR Radiofrequency Ablation OR Radio Frequency Ablation OR Radio-frequency Ablation)

5- #1 AND #2 AND #3 AND #4

No limitations were applied.

Filed of search: All fields’

From inception to July 31, 2023

Results: **206**

**Cochrane:**

1-(Liver neoplasm* OR Hepatic Neoplasm* OR Hepatocellular Cancer* OR Liver Cancer* OR Hepatic Cancer* OR Hepatocellular Carcinoma* OR HCC OR Liver Cancer OR Liver cell Carcinoma* OR Hepatoma* OR hepatic malignanc*)

2- (Laparoscopic OR Laparoscop* OR Celioscop* OR Peritoneoscop* OR Laparoscopic Surgery)

3- (Hepatectomy AND Hepatectomies)

4- (Radiofrequency OR Radiofrequency Ablation OR Radio Frequency Ablation OR Radio-frequency Ablation)

5- #1 AND #2 AND #3 AND #4

No limitations were applied.

From inception to July 31, 2023

Filed of search: All fields

Results: **17**

**Web of Science:**

1- ALL=(Liver neoplasm* OR Hepatic Neoplasm* OR Hepatocellular Cancer* OR Liver Cancer* OR Hepatic Cancer* OR Hepatocellular Carcinoma* OR HCC OR Liver Cancer OR Liver cell Carcinoma* OR Hepatoma* OR hepatic malignanc*)

2- ALL= (Laparoscopic OR Laparoscop* OR Celioscop* OR Peritoneoscop* OR Laparoscopic Surgery)

3- ALL=(Hepatectomy AND Hepatectomies)

4- ALL=(Radiofrequency OR Radiofrequency Ablation OR Radio Frequency Ablation OR Radio-frequency Ablation)

5- ALL= (#1 AND #2 AND #3 AND #4)

No limitations were applied.

From inception to July 31, 2023

Results: **12**

**Scopus:**

1-TITLE-ABS-KEY (“Liver neoplasm* “OR” Hepatic Neoplasm*” OR “Hepatocellular Cancer*” OR “Liver Cancer*” OR” Hepatic Cancer*” OR” Hepatocellular Carcinoma*” OR HCC OR” Liver Cancer “OR “Liver cell Carcinoma*” OR Hepatoma* OR “hepatic malignanc*”)

2-TITLE-ABS-KEY (Laparoscopic OR Laparoscop* OR Celioscop* OR Peritoneoscop* OR Laparoscopic Surgery)

3- TITLE-ABS-KEY(Hepatectomy AND Hepatectomies)

4- TITLE-ABS-KEY(Radiofrequency OR” Radiofrequency Ablation” OR” Radio Frequency Ablation “OR “Radio-frequency Ablation”)

5-TITLE-ABS-KEY (#1 AND #2 AND #3 AND #4)

No limitations were applied.

From inception to July 31, 2023

Results: **292**

Supplementary figures:

**Supplementary figure S1: Sensitivity analysis of overall survival at 3 years**


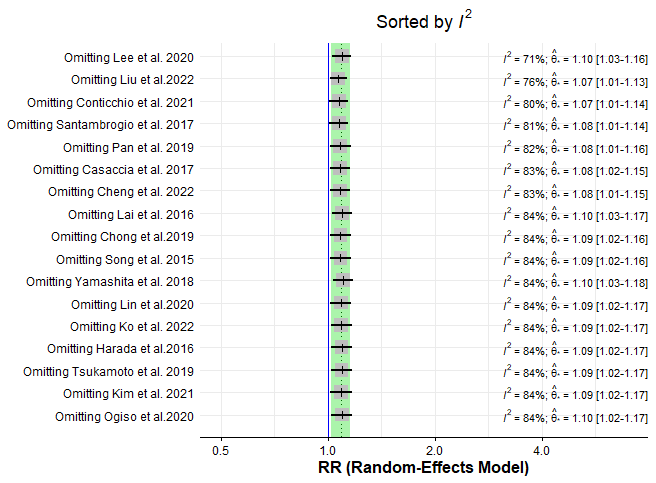


**Supplementary figure S2: Sensitivity analysis of overall survival at 5 years**


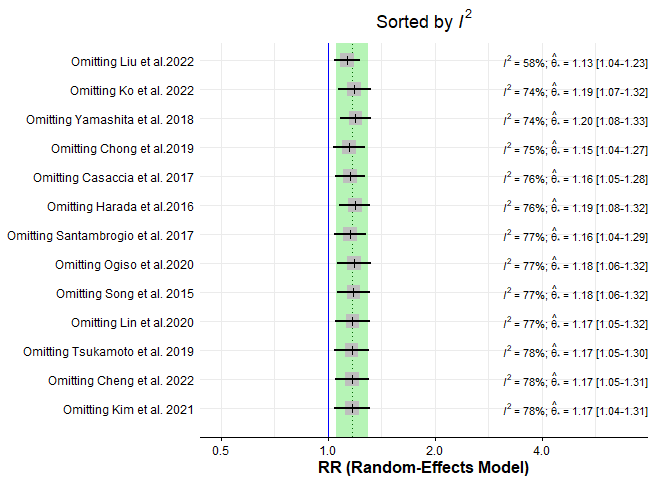


**Supplementary file figure S3: Forrest plot illustrating subgroup analysis for 1-year overall survival based on RFA type**


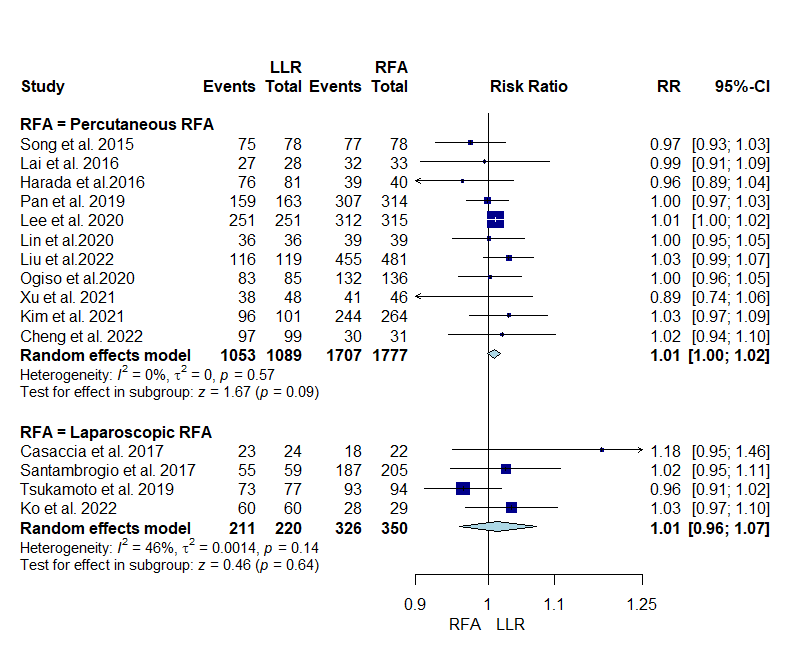


**Supplementary file figure S4: Forrest plot illustrating Subgroup analysis for 3-years overall survival based on RFA type**


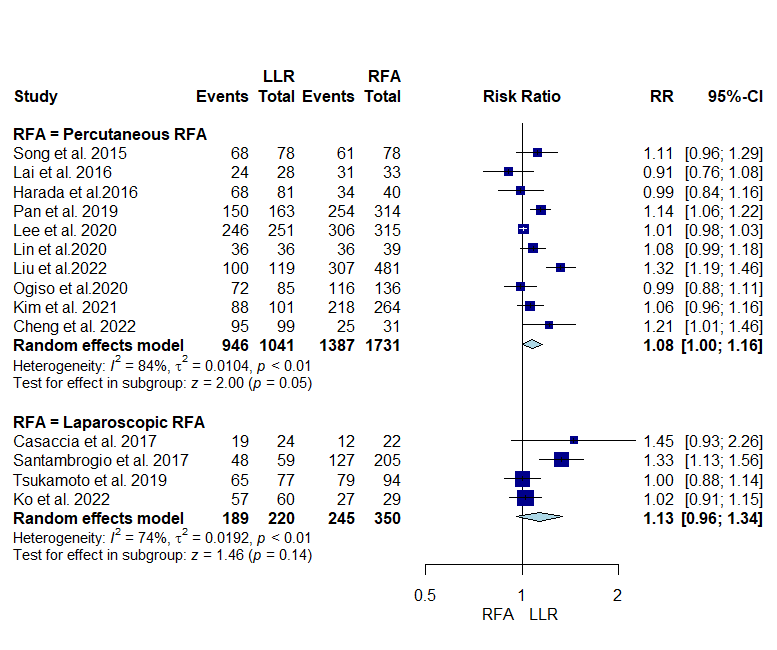


**Supplementary file figure S5: Sensitivity analysis of laparoscopic subgroup overall survival at 3 years**

**
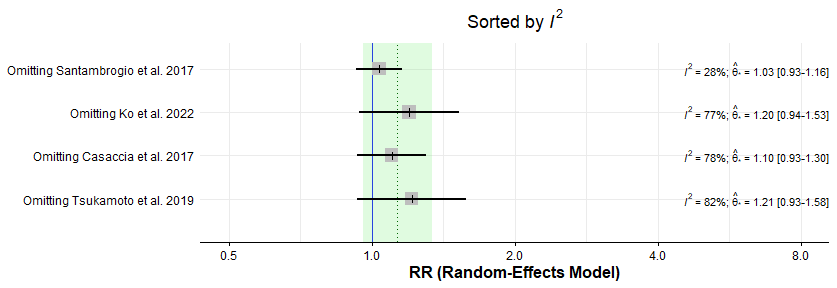
**

**Supplementary file figure S6: Sensitivity analysis of percutaneous subgroup overall survival at 3 years
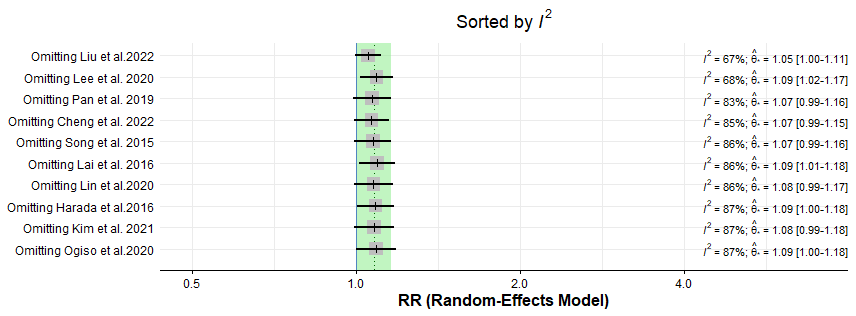
**

**Supplementary file figure S7:Forrest plot illustrating Subgroup analysis for 5-years overall survival based on RFA type**

**
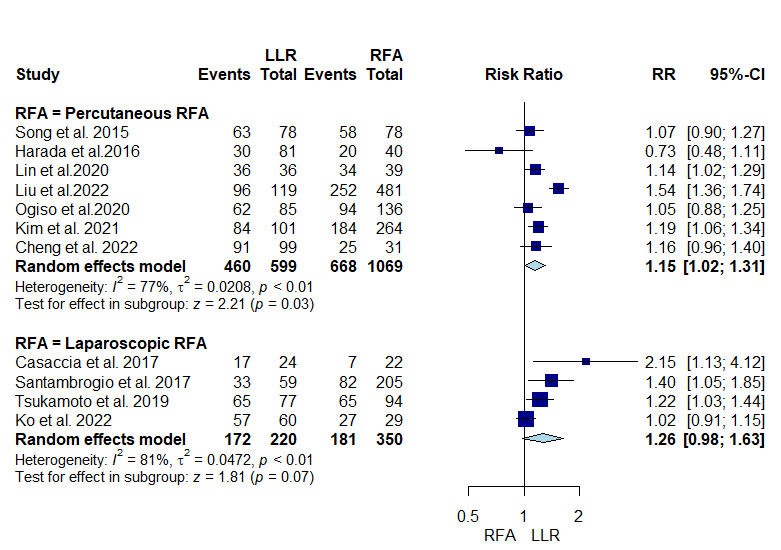
**

**Supplementary file figure S8 :Sensitivity analysis of percutaneous subgroup overall survival at 5 years**

**
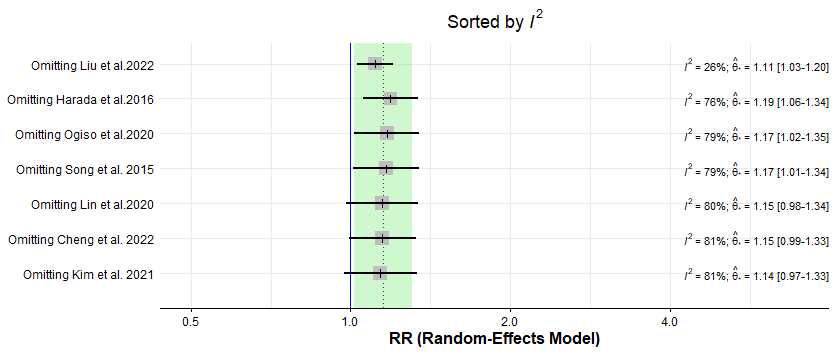
**

**Supplementary file figure S9:Sensitivity analysis of laparoscopic subgroup overall survival at 5 years**

**
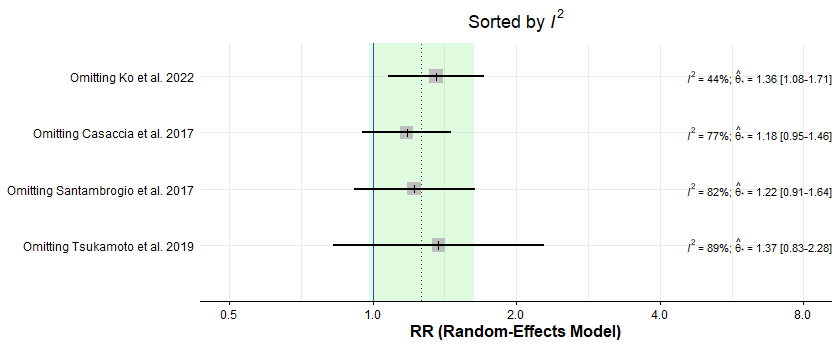
**

**
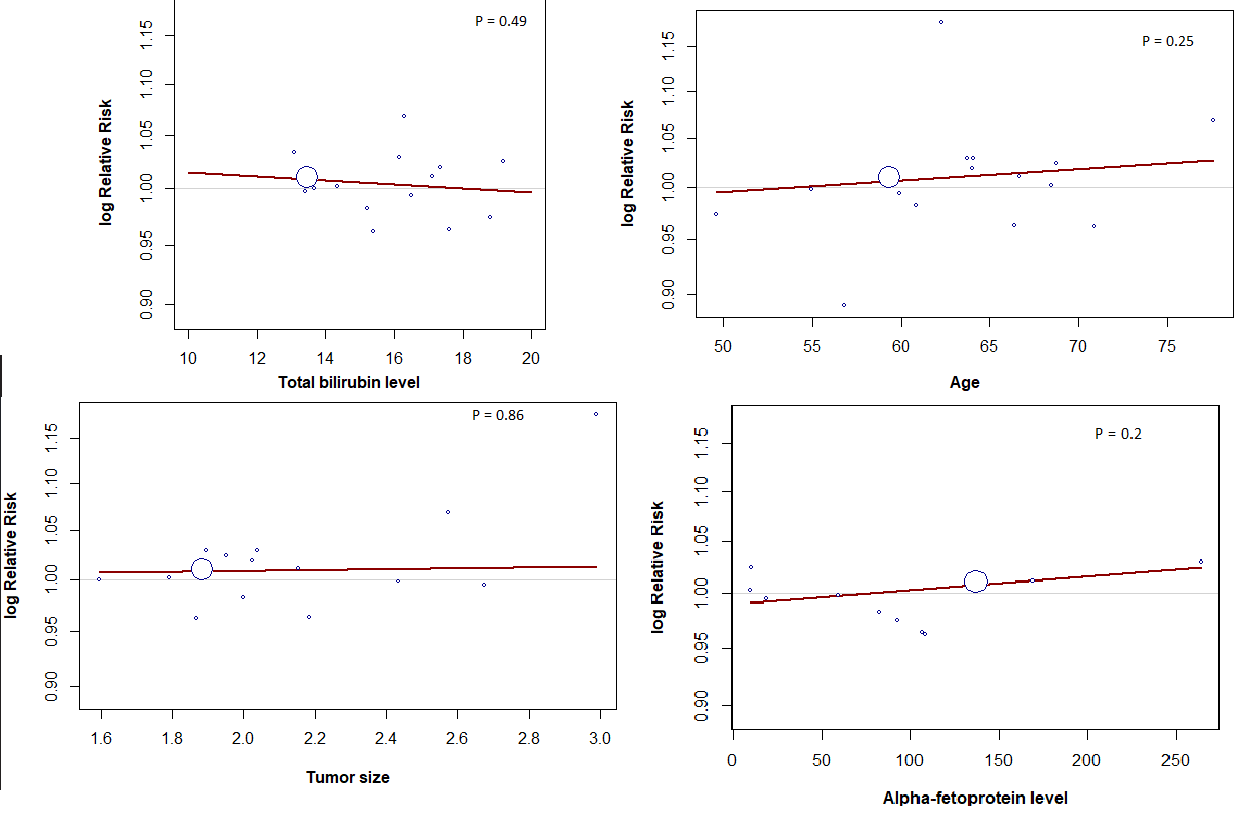
Supplementary file figure S10: Meta-Regression Analysis of Covariates and 1-Year overall Survival**

**Supplementary file figure S11:Forrest plot illustrating overall survival PSM**

**
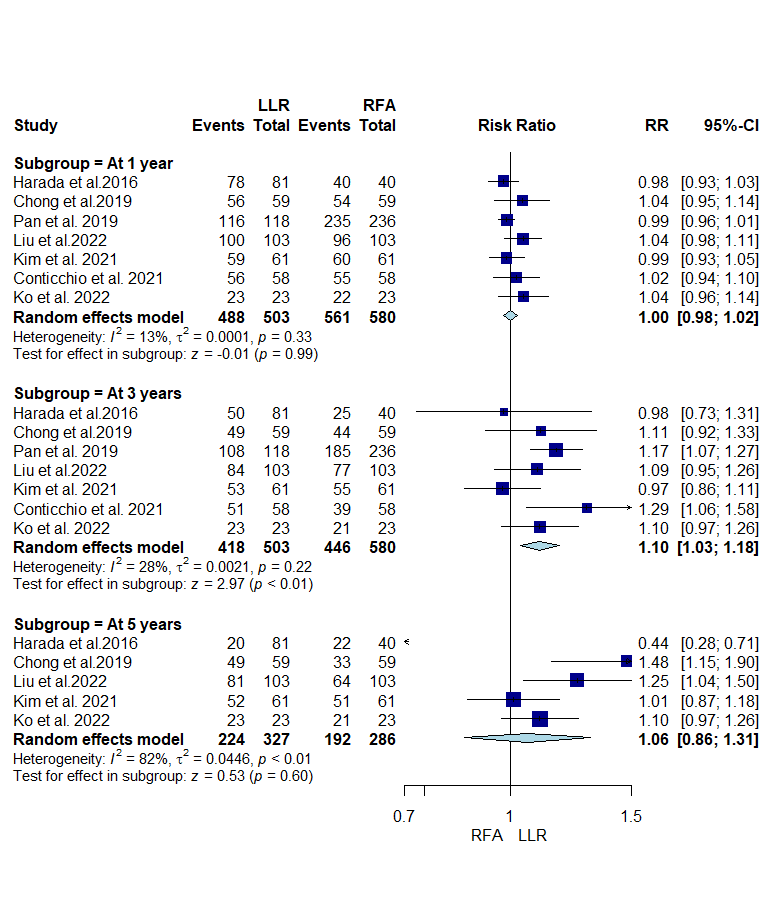
**

**Supplementary file figure S12: Sensitivity analysis of overall survival PSM at 5 years**

**
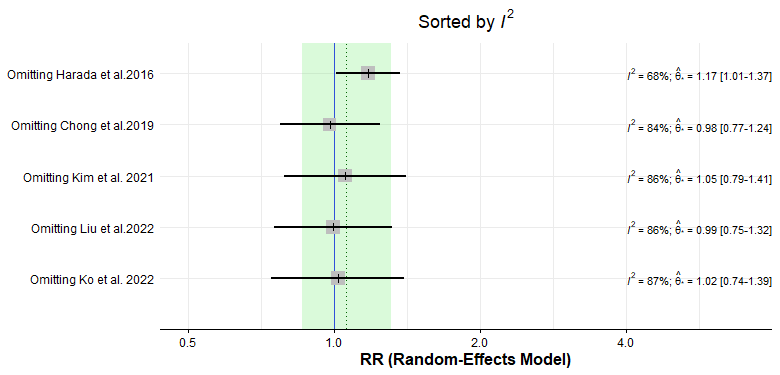
**

**Supplementary** **file Figure S13:Sensitivity analysis of disease-free survival at 3 years**

**
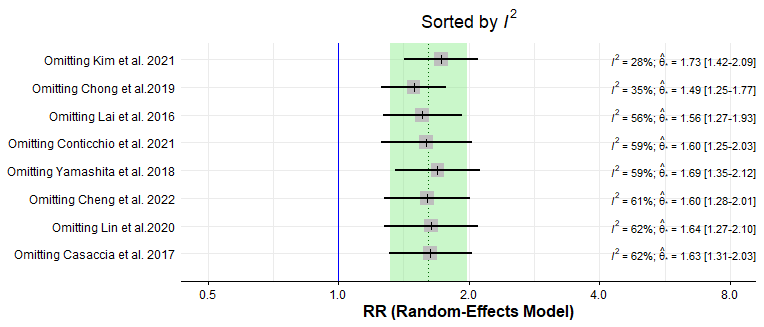
**

**Supplementary file Figure S14:Sensitivity analysis of disease-free survival at 1 year**

**
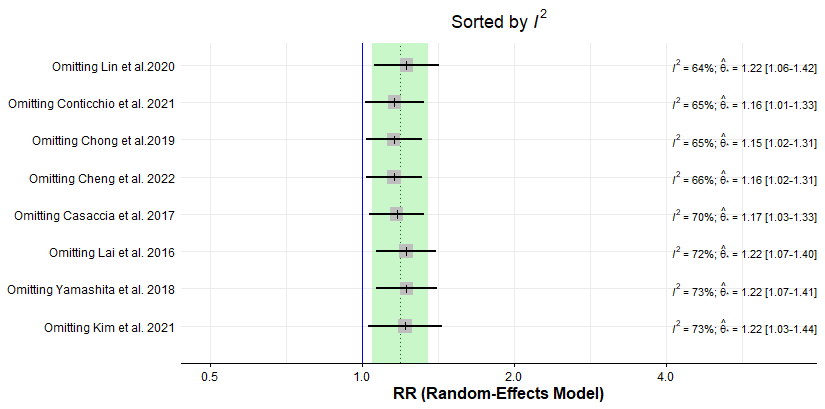
**

**Supplementary file Figure S15:Sensitivity analysis of Disease-free survival at 5 years
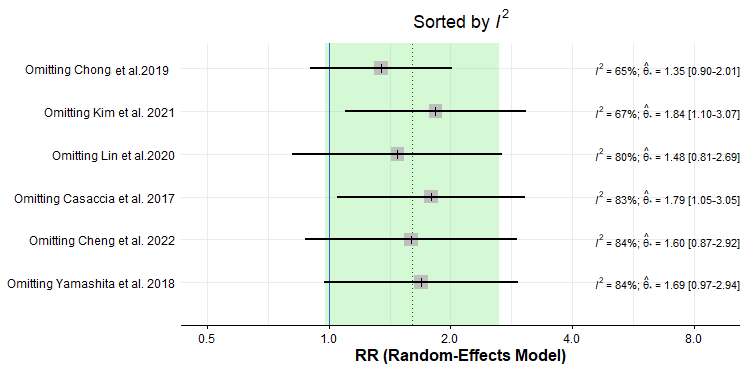
**

**Supplementary file Figure S16: Forrest plot illustrating disease-free survival PSM**


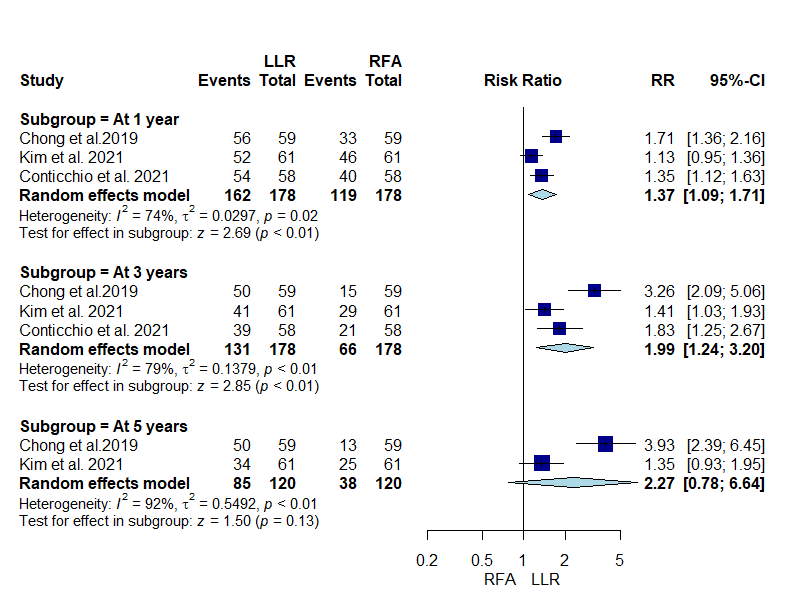


**Supplementary file Figure S17:Sensitivity analysis of disease-free survival PSM at 1 year**

**
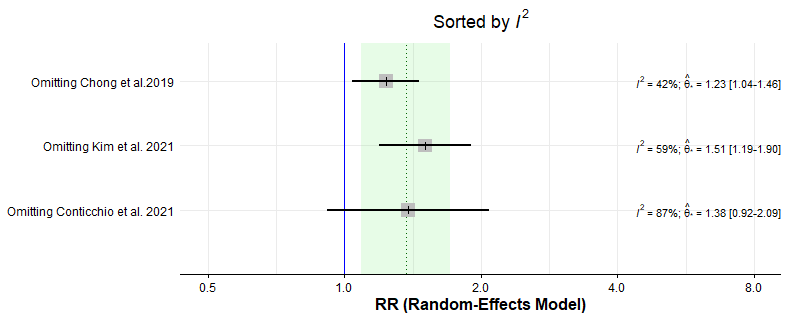
**

**Supplementary file Figure S18: Sensitivity analysis of disease-free survival PSM at 3 years**

**
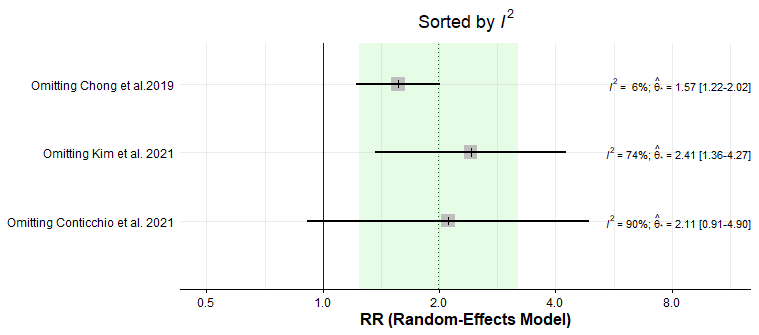
**

**Supplementary file Figure S19: Sensitivity analysis of recurrence-free survival at 1 year**

**
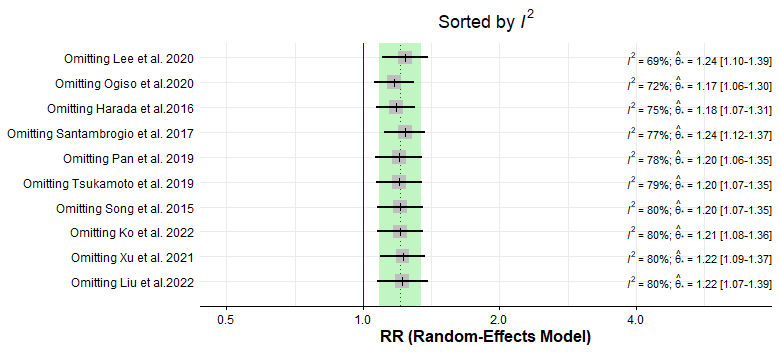
**

**Supplementary file Figure S20:Sensitivity analysis of recurrence-free survival at 3 years
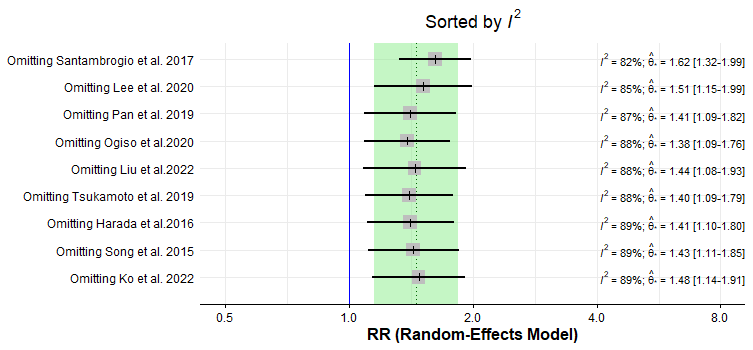
**

**Supplementary file Figure S21:Sensitivity analysis of recurrence-free survival at 5 years**

**
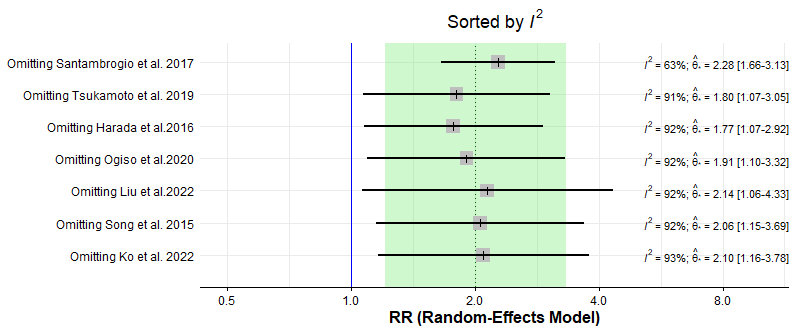
**

**Supplementary file Figure S22: Forrest plot illustrating subgroup analysis for 1-year recurrence-free survival based on RFA type**

**
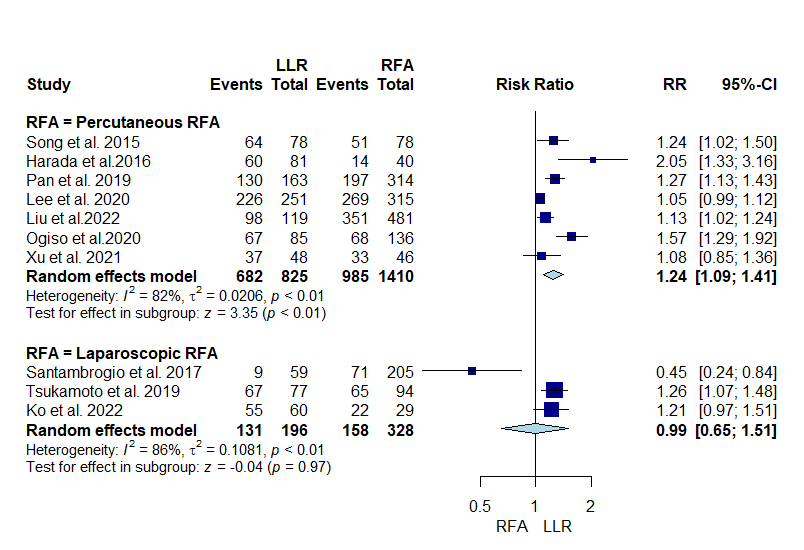
**

**Supplementary file Figure S23: Forrest plot illustrating subgroup analysis for 3-years recurrence-free survival based on RFA type
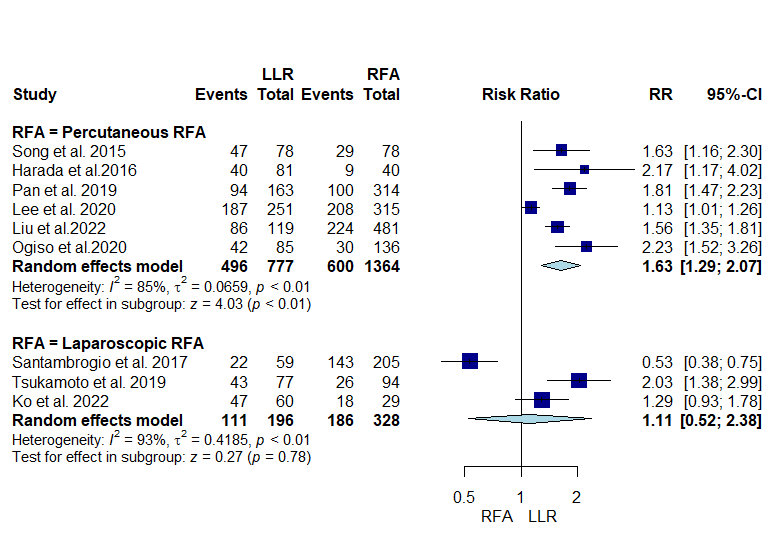
**

**Supplementary file Figure S24: Sensitivity analysis of percutaneous subgroup recurrence free survival at 1 year**

**
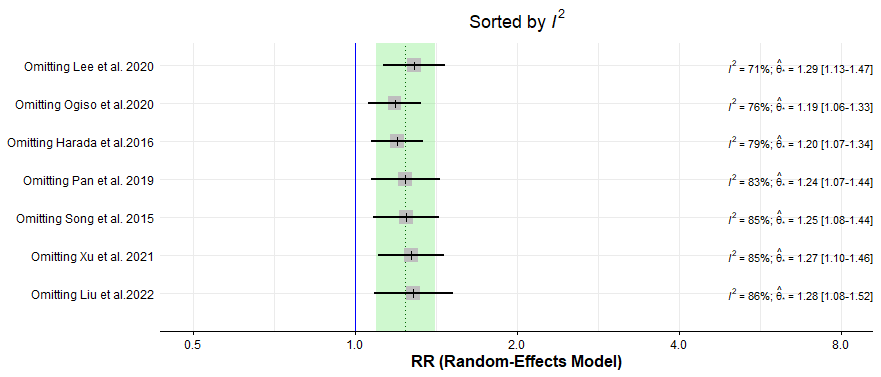
**

**Supplementary file Figure S25:Sensitivity analysis of percutaneous subgroup recurrence free survival at 3 years**

**
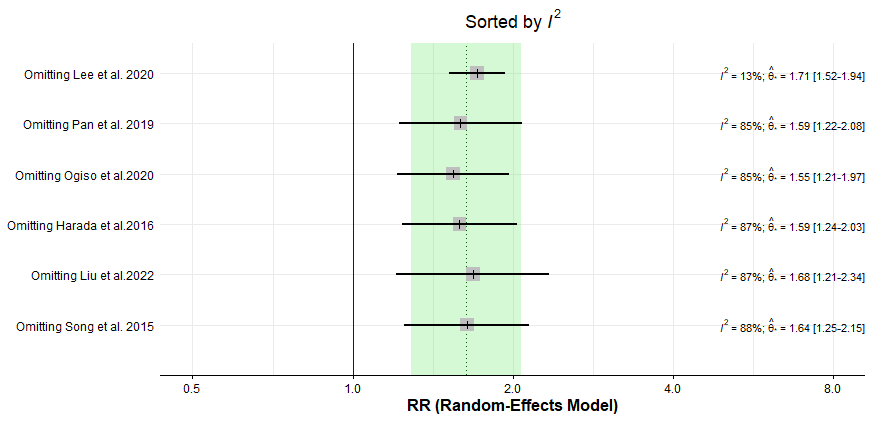
**

**Supplementary file Figure S26 :Sensitivity analysis of laparoscopic subgroup recurrence free survival at 1 year**

**
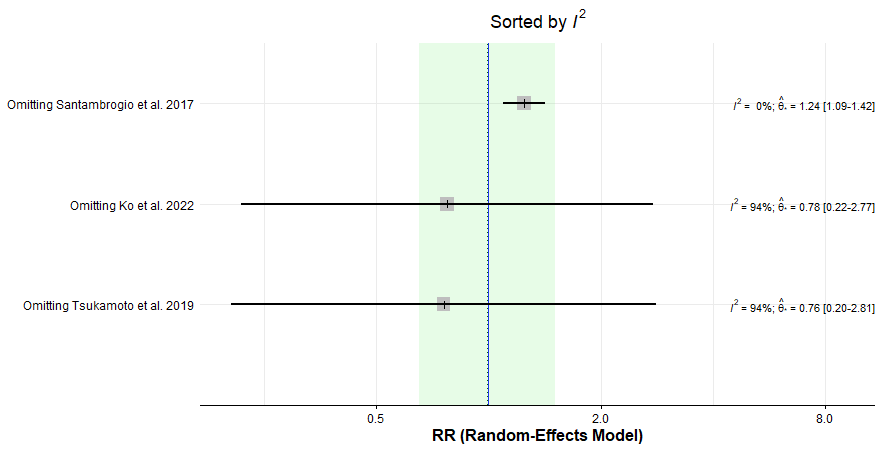
**

**Supplementary file Figure S27 :Sensitivity analysis of laparoscopic subgroup recurrence free survival at 3 years**

**
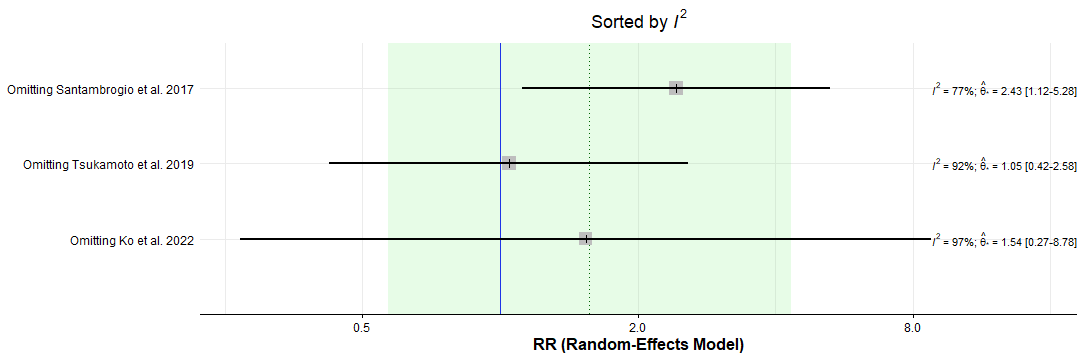
**

**Supplementary file Figure S28; Forrest plot illustrating Subgroup analysis for 5-years Recurrence-free survival based on RFA type**

**
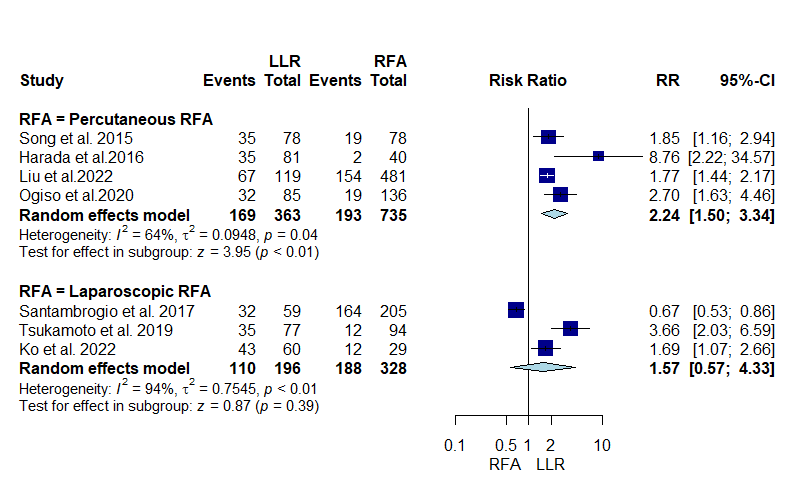
**

**Supplementary file Figure S29:Sensitivity analysis of percutaneous subgroup recurrence free survival at 5 years
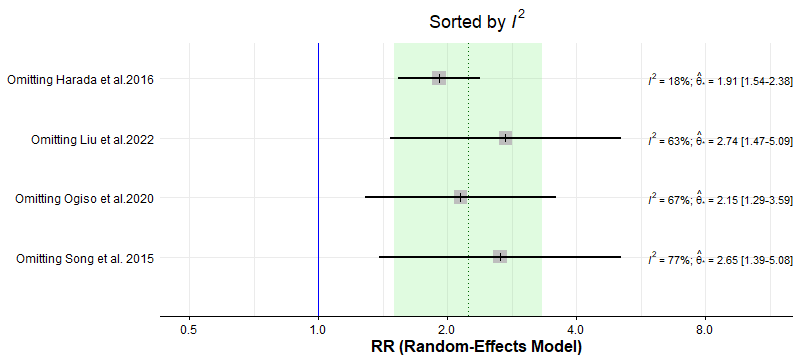
**

**Supplementary file Figure S30: Sensitivity analysis of laparoscopic subgroup recurrence free survival at 5 years
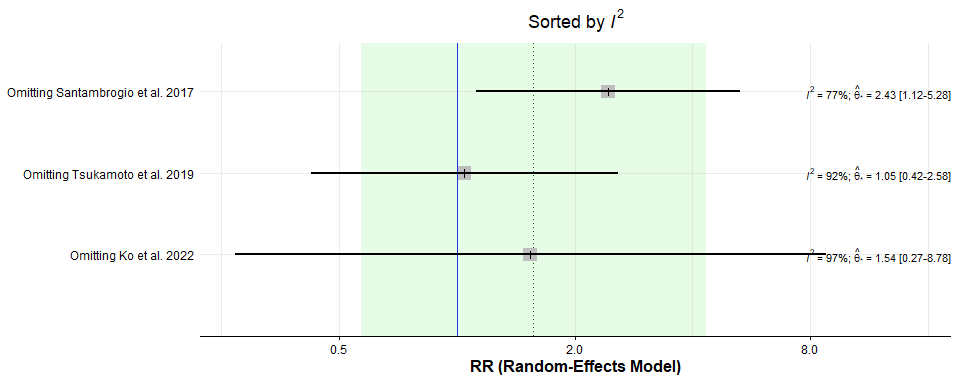
**

**Supplementary file Figure S31: Forrest plot illustrating recurrence-free survival PSM**

**
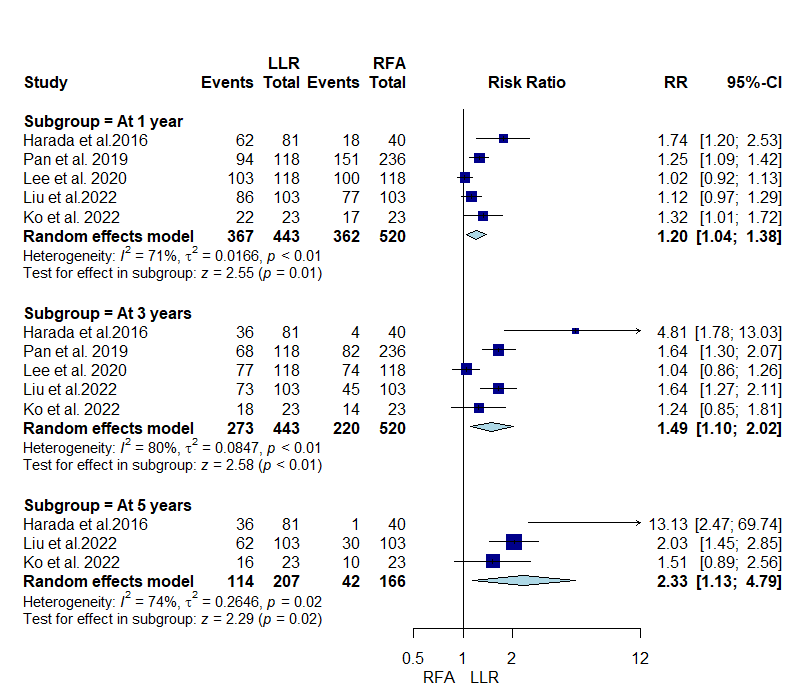
**

**Supplementary file Figure S32:Sensitivity analysis of recurrence-free survival PSM at 1 year**

**
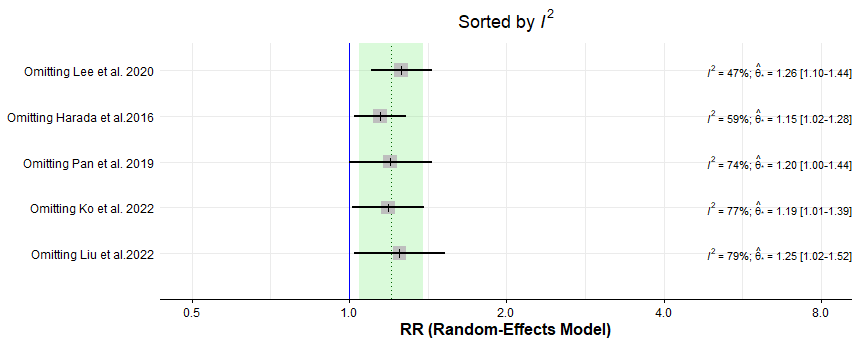
**

**Supplementary file Figure S33: Sensitivity analysis of recurrence-free survival PSM at 5 years**

**
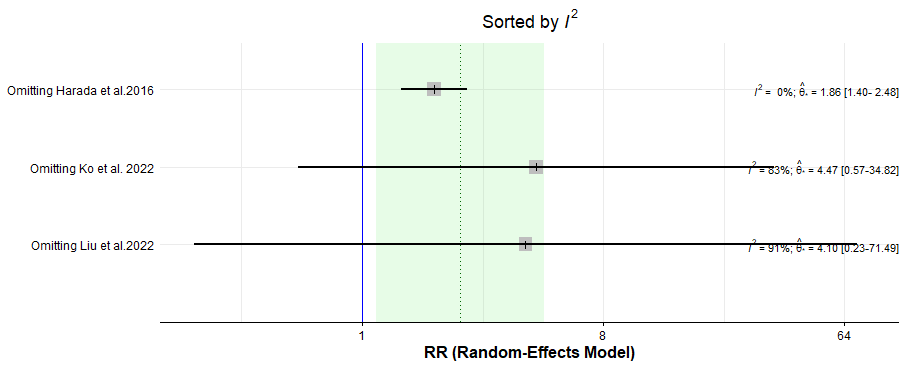
**

**Supplementary file Figure S34: Sensitivity analysis of recurrence-free survival PSM at 3 years**

**
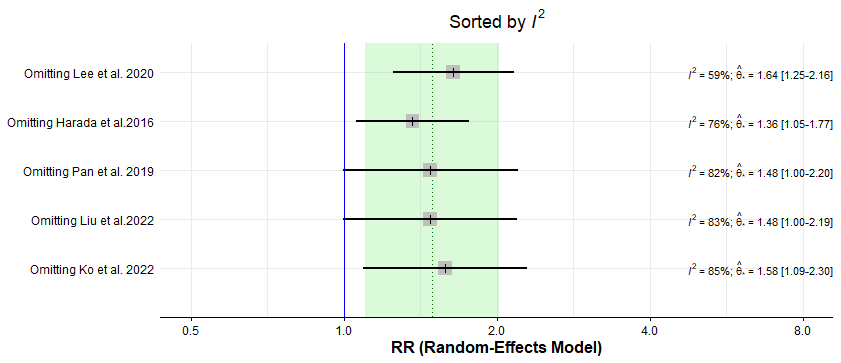
**

**Supplementary file Figure S35:Sensitivity analysis of local recurrence**

**
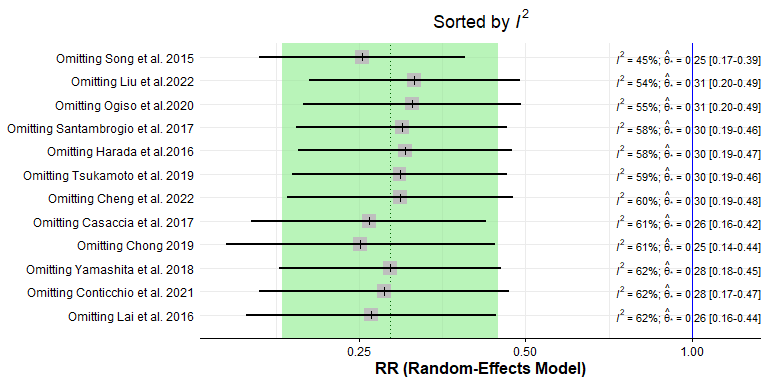
**

**Supplementary file Figure S36: Forrest plot illustrating Subgroup analysis for local recurrence based on RFA type**

**
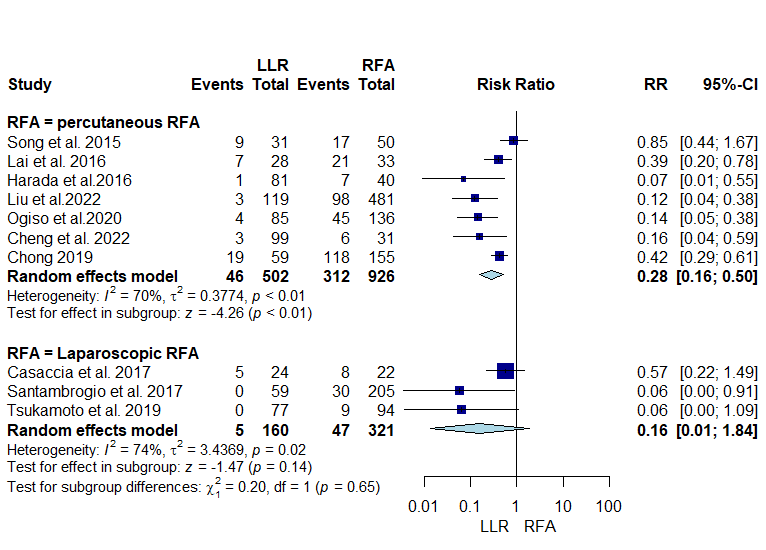
**

**Supplementary file Figure S37:Sensitivity analysis of percutaneous subgroup local recurrence**

**
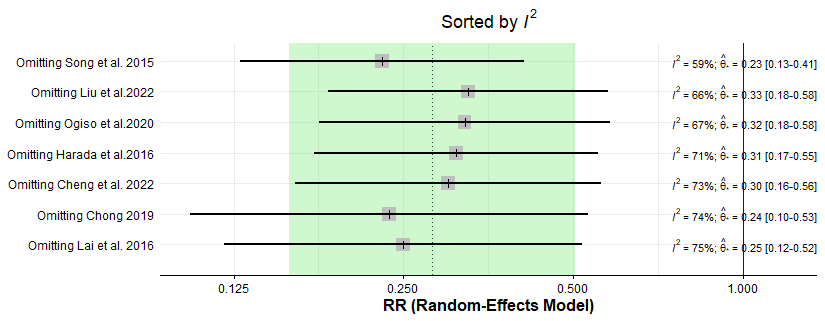
**

**Supplementary file Figure S38:Sensitivity analysis of laparoscopic subgroup local recurrence**

**
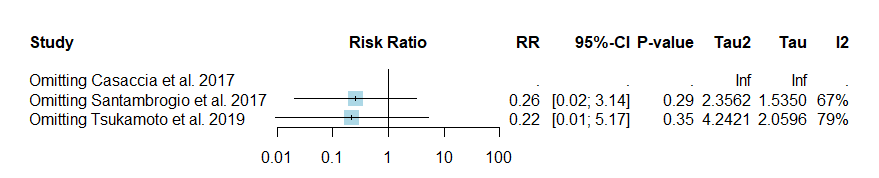
**

**Supplementary file Figure S39: Forrest plot illustrating intrahepatic recurrence**

**
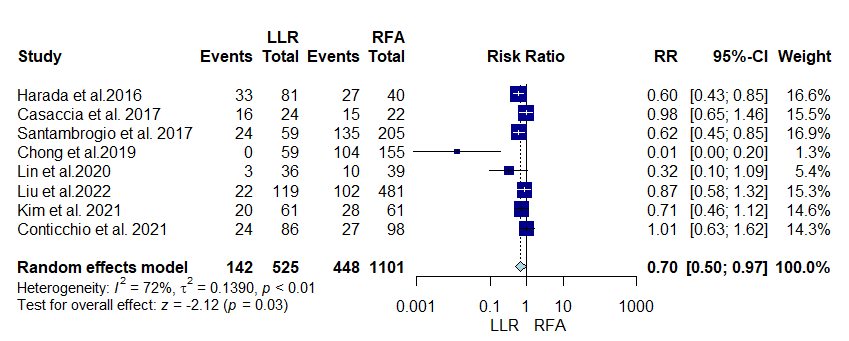
**

**Supplementary file Figure S40:Sensitivity analysis of intrahepatic recurrence**

**
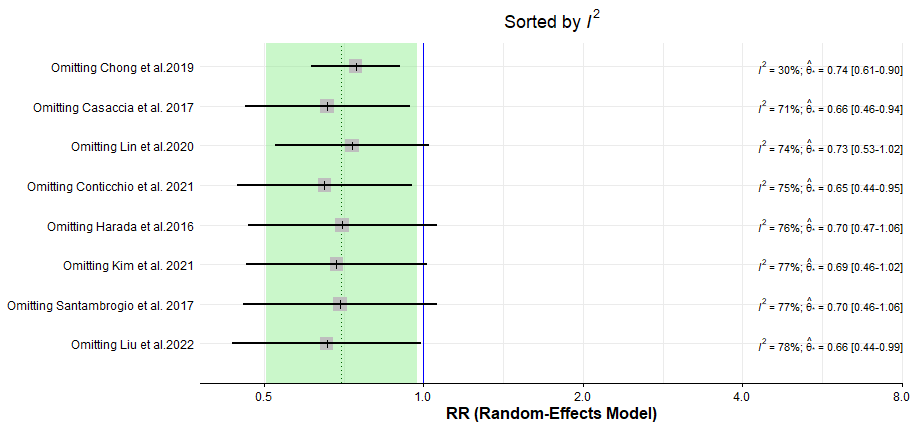
**

**Supplementary file Figure S41:Forrest plot illustrating extrahepatic recurrence**

**
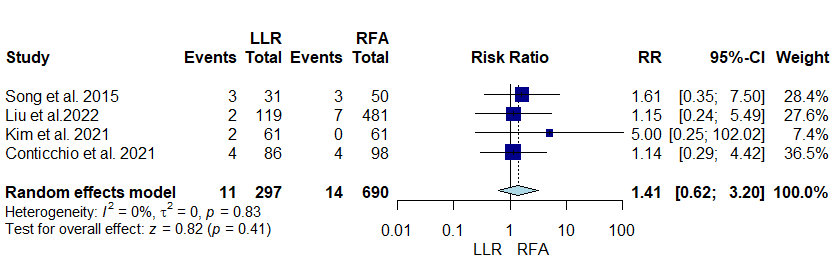
**

**Supplementary file Figure S42:Forrest plot illustrating duration of surgery**

**
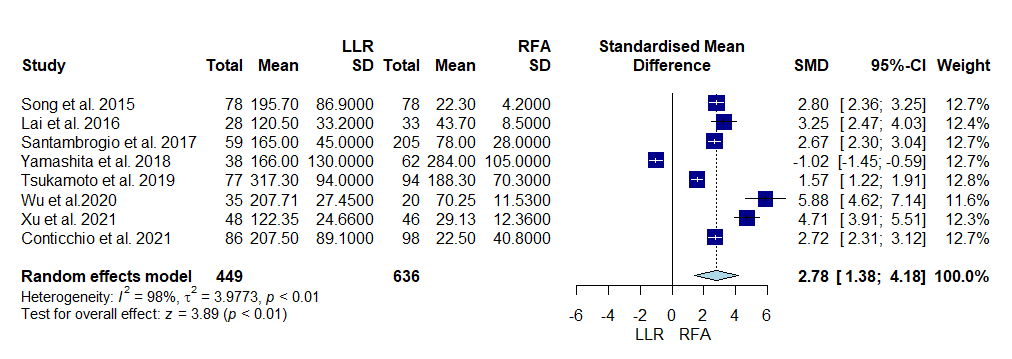
**

**Supplementary file Figure S43:Sensitivity analysis of duration of surgery**

**
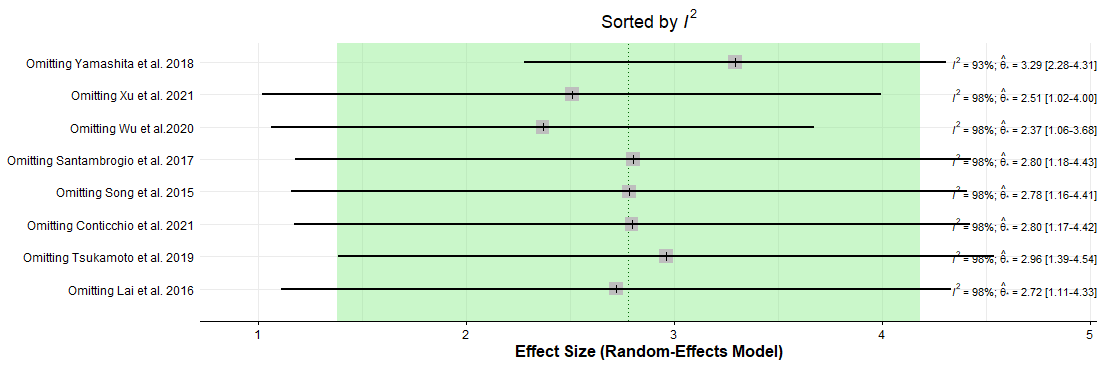
**

**Supplementary file Figure S44: Forrest plot illustrating incidence of blood transfusion during surgery**

**
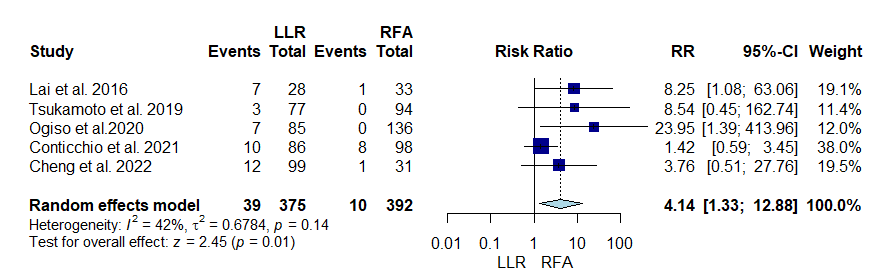
**

**Supplementary file Figure S45:Forrest plot illustrating all complications**

**
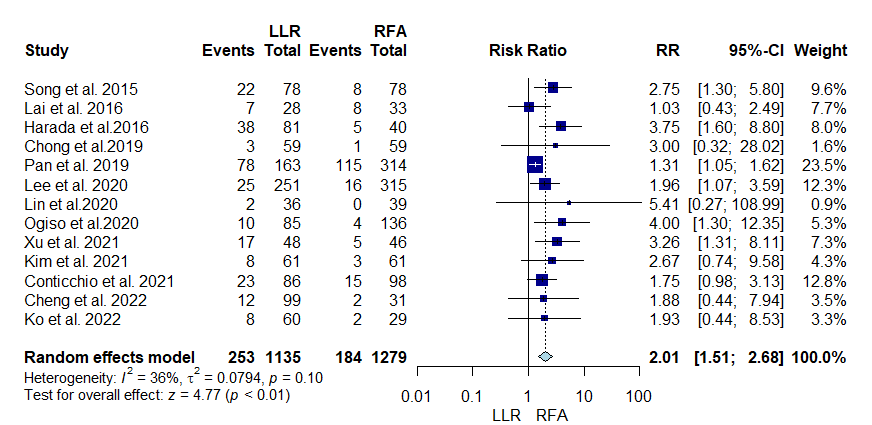
**

**Supplementary file Figure S46:Forrest plot illustrating 90-days mortality**

**
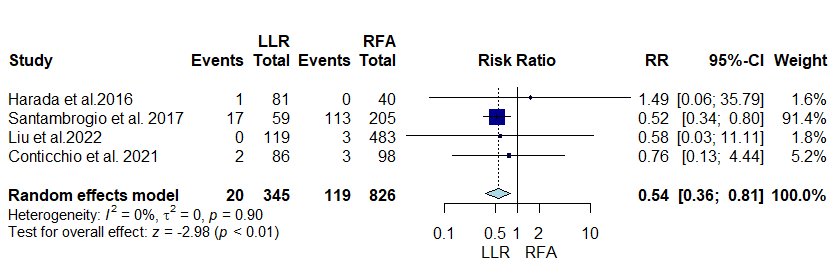
**

**Supplementary file Figure S47:Forrest plot illustrating 30-days mortality**

**
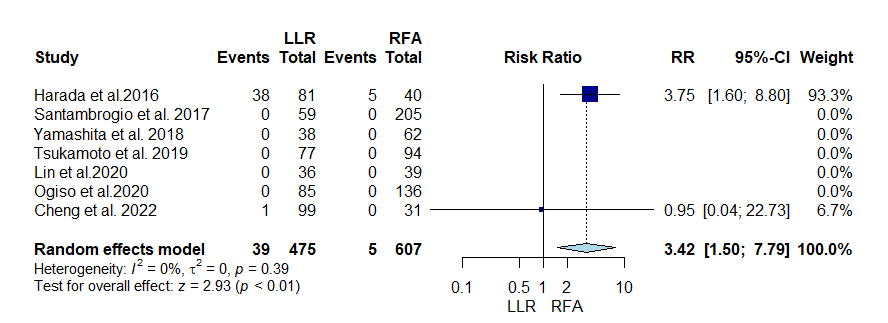
**

**Supplementary file Figure S48:Forrest plot illustrating major complications**

**
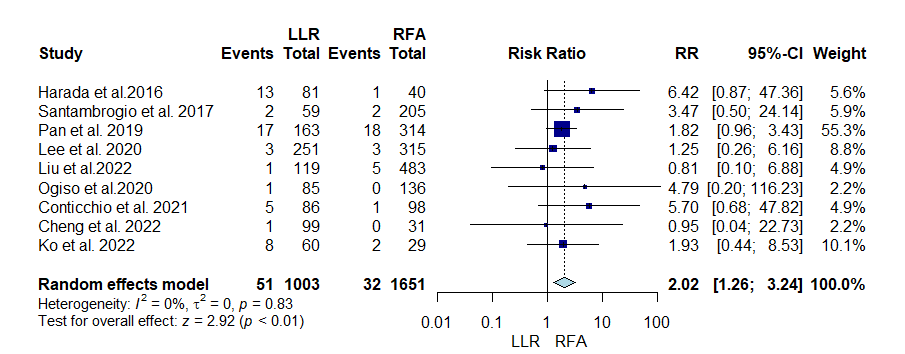
**

**Supplementary file Figure S49:Forrest plot illustrating duration of hospital stay**

**
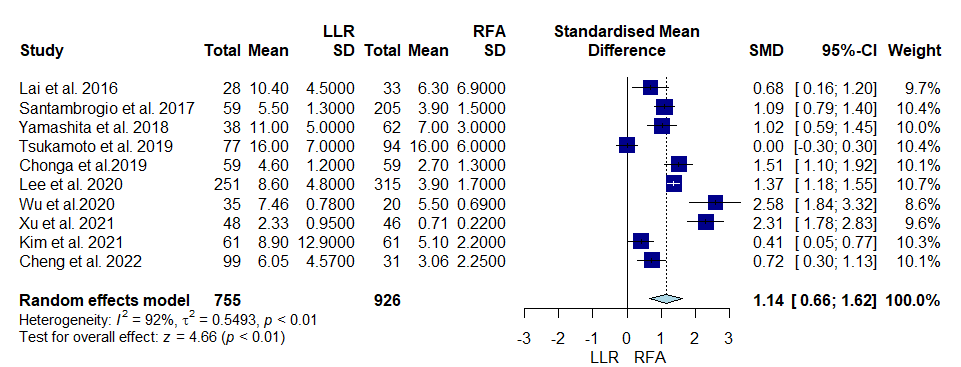
**

**Supplementary file Figure S50:Sensitivity analysis of duration of hospital stay**


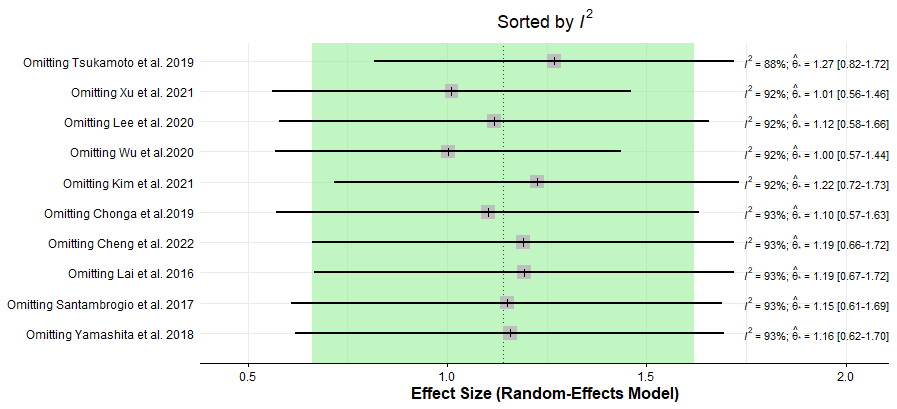


**Supplementary file Figure S51:Funnel plot for the local recurrence**
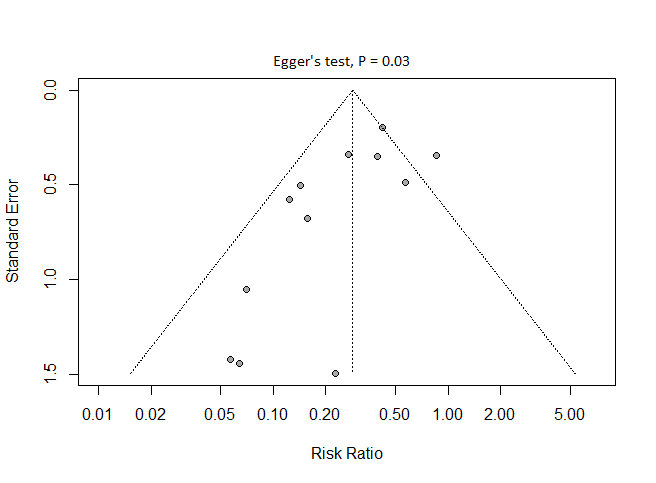


**Supplementary file Figure S52:Funnel plot (trim and fill method) for the local recurrence**

**
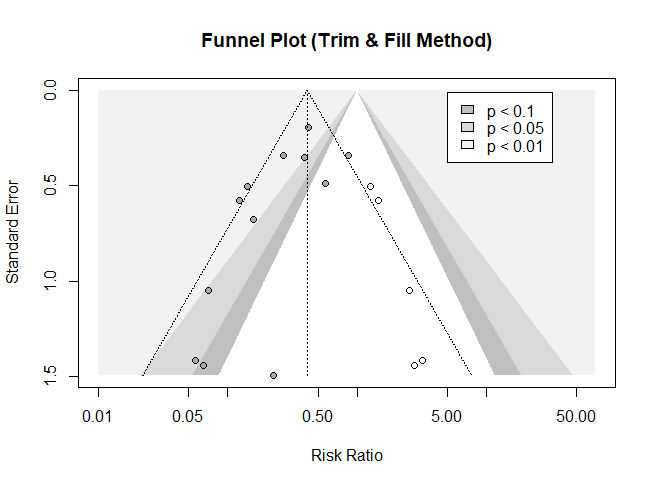
**
