# Supplementary material for: Efficacy and safety of laparoscopic liver resection versus radiofrequency ablation in patients with early and small hepatocellular carcinoma: an updated meta-analysis and meta-regression of observational studies
Source: World J Surg Oncol. 2024 Feb 7;22:47. doi: 10.1186/s12957-023-03292-3 (PMC10848480; doi:10.1186/s12957-023-03292-3)
Supplement: Supplementary file 2 — Additional file 2: Supplementary Table 1. Baseline characteristics of enrolled patients in each included study. [file 12957_2023_3292_MOESM2_ESM.docx]

| **Study ID** | **Groups** | **Number of patients** | **Age** | **Males**  **n (%)** | **Maximal tumor size**  **(cm)** | **Platelet count** | **Total bilirubin level,** μ**mol/L** | **Alpha-Fetoprotein level, ng/mL** | **HBV**  **n (%)** | **HCV**  **n (%)** | **ALCOHOLIC**  **n (%)** | **Tumor number**  **solitary**  **n (%)** | **Tumor number**  **Multiple**  **n (%)** |
| --- | --- | --- | --- | --- | --- | --- | --- | --- | --- | --- | --- | --- | --- |
| ^song 2015^  (30) | LLR | 78 | 48 [44, 57] | 70 (90 %) | N/A | 115.9 ± 48.2  (/L 10^9^ x) | 17.9 ± 5.7 | 38.5 [6.9, 281.9] | 73 (94 %) | N/A | N/A | N/A | N/A |
|  | RFA | 78 | 48 [43. 58] | 70 (90 %) | N/A | 106.0 ± 39.1  (/L 10^9^ x) | 19.7 ± 8.4 | 43.0 [6.0, 181.7] | 77 (99 %) | N/A | N/A | N/A | N/A |
| ^LAI 2016^  (36) | LLR | 28 | 56.5±12.6 | 24 (86%) | 3.0±1.1 | 142.2±51.6  (/L 10^9^ x) | 14.5±5.8 | 15.5 (1.7–16932.7) | 23 (82%) | 1 (4%) | 12 (43%) | 26(93%) | 2(7%) |
|  | RFA | 33 | 62.8±11.3 | 29 (88%) | 2.4±0.9 | 110.8±46.5  (/L 10^9^ x) | 18.2±11.4 | 21.7 (1.9–4097.0) | 25 (76%) | 1 (3%) | 9 (27%) | 30(91%) | 3(9%) |
| ^Harada 2016^  (38) | LLR | 81 | 70.4 (53–85) | 45 (55.6 %) | 2.1 (0.7–5) | 10.3 (4.6–24)  ((10^4^ /μL | 13.68 (3.42-39.33) | 132 (2.8–2340) | 8 (10 %) | 61 (75 %) | 9 (11 %) | N/A | N/A |
|  | RFA | 40 | 71.9 (58–87) | 23 (57.5 %) | 1.4 (0.7–2.4) | 9.7 (3.8–33)  ((10^4^ /μL | 18.81(6.84-41.04) | 61 (3.3–283) | 2 (5 %) | 29 (73 %) | 4 (10 %) | N/A | N/A |
| ^Casaccia 2017^  (15) | LLR | 24 | 63.58 ± 9.55 | 16 (67%) | 3.3 ±1.383 | N/A | N/A | N/A | 6 (25%) | 11 (46%) | 6 (25%) | 20 (83%) | 4 (16%) |
|  | RFA | 22 | 60.82 ± 7.25 | 18 (75%) | 2.625 ±1.313 | N/A | N/A | N/A | 8 (36%) | 8 (36%) | 3 (14%) | 6 (27%) | 16 (73%) |
| ^Santambrogio 2017^  (31) | LLR | 59 | 68 ± 9 | 42 (71%) | 2.09 ±0.67 | 123 ± 55 (×10^3^/mm^3^) | 19.84±8.55 | 7.5 [3.7–17.2] | 8 (14%) | 43 (72%) | N/A | N/A | N/A |
|  | RFA | 205 | 69 ± 9 | 152 (74%) | 1.91 ±0.58 | 112 ± 55 (×10^3^/mm^3^) | 18.98±8.55 | 6.75 [3.2–23.5] | 29 (14%) | 136 (66%) | N/A | N/A | N/A |
| ^Yamashita 2018^  (27) | LLR | 38 | 66.9±9.1 | 25 (66%) | 2.4±0.9 | N/A | 17.1±10.26 | 291±1320 | 5 (13%) | 30 (79%) | N/A | 32(84%) | 6(16%) |
|  | RFA | 62 | 66.5±9.5 | 40 (65%) | 2.0±0.6 | N/A | 17.1±8.55 | 94±202 | 9 (15%) | 45 (73%) | N/A | 42(68%) | 20(32%) |
| ^Tsukamoto 2019^  (29) | LLR | 77 | 65.2±10.2 | 53 (69%) | 2.25 ± 0.9 | 14.4± 5.3  ((10^4^ /μL | 14.71±7.01 | 167.6±516.6 | 22 (29%) | 35 (46%) | N/A | N/A | N/A |
|  | RFA | 94 | 67.4±8.1 | 51 (54%) | 2.13 ± 0.66 | 9.7± 4.7  ((10^4^ /μL | 19.98±9.41 | 56.3±172.0 | 12 (13%) | 64 (68%) | N/A | N/A | N/A |
| ^Chong 2019^  (39) | LLR | 59 | 57.7±10.5 | 46 (78%) | 2.0 [1.6–2.8] | 150.4±62.2  (/L 10^9^ x) | 11.6±5.6 | 59 [4.0–436.0] | 48 (81%) | 4 (7%) | N/A | 56 (95%) | 3(5%) |
|  | RFA | 155 | 62.1±9.8 | 120 (77%) | 2.0 [1.6–2.7] | 129.1±66.9  (/L 10^9^ x) | 16.6±10.8 | 17.0 [6.0–129.0] | 118 (76%) | 23 (15%) | N/A | 146 (94%) | 9(6%) |
| ^Pan 2019^  (32) | LLR | 163 | 51.00 [44.00-60.00] | 140 (86 %) | 2.50 [2.00 - 4.00] | N/A | 14.1±6.03 | 19.50 [4.42-218.00] | 6 (4%) | N/A | N/A | 138 (85%) | 25 (15%) |
|  | RFA | 314 | 57.00 [46.00-65.00] | 273 (87 %) | 2.40 [1.80 -3.20] | N/A | 13.05±4.97 | 7.27 [3.14 -134.00] | 54 (17%) | N/A | N/A | 265 (84%) | 49 (16%) |
| ^Lee 2020^  (44) | LLR | 251 | 57.5±9.3 | 199 (79 %) | 2.13±1.44 | 168.4±53.5 K/mm3 | 13±6.33 | 218.4±904.2 | 196 (78%) | 21 (8%) | 29 (12%) | N/A | N/A |
|  | RFA | 315 | 60.8±9.6 | 227 (88%) | 1.69±0.50 | 130.2±50.9 K/mm3 | 13.85±7.52 | 72.9±211.6 | 234 (74%) | 34 (11%) | 40 (13%) | N/A | N/A |
| ^Lin 2020^  (35) | LLR | 36 | N/A | 27(75%) | 1.7±0.25 | 167.5±34.61 (k/μl) | 13.68±4.1 | 1118.4 (±2512.31) IU/L | 25 (69%) | 9 (25%) | N/A | N/A | N/A |
|  | RFA | 39 | N/A | 25 (64 %) | 1.5±0.22 | 170.5±56.81 (k/μl) | 13.68±6.16 | 172.7 (±345.81) IU/L | 25 (64.10% | 17 (43.59%) | N/A | N/A | N/A |
| ^Ogiso 2020^  (33) | LLR | 85 | 69 (46–88) | 62 (73%) | 2.1 (0.8–0.3) | 13.8 (4.1–75)  ((10^4^ /μL | 15.39(1.71-44.46) | 8.1 (1.7–1915) | 20 (24%) | 47 (55%) | 6 (7%) | 73 (86 %) | 12 (14 %) |
|  | RFA | 136 | 73 (47–87 | 98 (26%) | 1.6 (0.5–0.3) | 10.9 (3.1-30.7)  ((10^4^ /μL | 13.68(5.13-35.91) | 10.8 (1.3–443.1) | 21 (15 %) | 85 (63%) | 15 (11%) | 115 (85%) | 21(15 %) |
| ^Wu 2020^  (42) | LLR | 35 | 61.8±8.51 | 30 | 3.56 ± 0.68 | N/A | N/A | 19 | 28(80%) | N/A | N/A | N/A | N/A |
|  | RFA | 20 | 61.6±6.67 | 17 | 3.50 ± 0.54 | N/A | N/A | 11 | 15(75%) | N/A | N/A | N/A | N/A |
| ^Xu 2021^  (28) | LLR | 48 | 57.24±13.23 | 39 (81%) | N/A | 106.41±34.06  (/L 10^9^ x) | N/A | N/A | 43 (90%) | N/A | N/A | N/A | N/A |
|  | RFA | 46 | 56.33±12.49 | 36 (78%) | N/A | 105.35±38.05  (/L 10^9^ x) | N/A | N/A | 42 (91 %) | N/A | N/A | N/A | N/A |
| ^Kim 2021^  (43) | LLR | 101 | 57.8 | 76 | 2.38 ±0.8 | 158.2±56  ((10^3^ /μL | 13.68±6.84 | 234.9±969.4 | 71 (70%) | 8 (8%) | 7 (7%) | N/A | N/A |
|  | RFA | 264 | 66.5 | 198 | 1.71 ±0.7 | 110.2±50  ((10^3^ /μL | 17.1±10.26 | 274.9±2496.3 | 183 (69%) | 31 (12%) | 23 (9%) | N/A | N/A |
| ^Conticchio 2021^  (40) | LLR | 86 | 75.7 (69.5–86.5) | 65 (76%) | 3 (1–3) | 179.5 (45–468)  (/L 10^9^ x) | 15.39(5.13-34.2) | N/A | 20 (23%) | 49 (57%) | 10 (12%) | N/A | N/A |
|  | RFA | 98 | 75 (70–89) | 65 (66%) | 2.2 (1–3) | 124 (10–856)  (/L 10^9^ x) | 17.1(3.42-42.75) | N/A | 6 (6%) | 56 (57%) | 17 (17%) | N/A | N/A |
| ^Cheng 2022^  (41) | LLR | 99 | 63.60±9.86 | 82 (83%) | 2.31±1.93 | 153.68±69.19  (/L 10^9^ x) | 17.27±6.97 | 47 [6.0–423.0] | 82 (83%) | 12 (12%) | N/A | 96 (97%) | 3 (3%) |
|  | RFA | 31 | 65.48±11.73 | 22 (71%) | 1.14±0.70 | 143.26±54.57  (/L 10^9^ x) | 17.61±8.77 | 34 [3.5–242.5] | 22 (71%) | 8 (26%) | N/A | 28 (90%) | 3 (10%) |
| ^Ko 2022^  (37) | LLR | 60 | 55.8 ± 9.0 | 42 (70%) | 2.1 (1.0–2.9) | 149 (68–368)  (/L 10^9^ x) | 11.97 (3.42-32.49) | 313.1 ± 823.2 | 42 (70%) | 4 (7%) | N/A | N/A | 57 (95%) |
|  | RFA | 29 | 60.0 ± 9.8 | 24 (83%) | 1.6 (1.0–2.8) | 109 (29–246)  (/L 10^9^ x) | 15.39 (3.42–35.91) | 13.9 ± 25.0 | 18 (62%) | 2 (7%) | N/A | N/A | 25 (86%) |
| ^Liu 2022^  (34) | LLR | 119 | 62[51.0–69.0] | 89 (74%) | 2.2[1.7–2.6] | N/A | N/A | N/A | 63 (53%) | 44 (37%) | 8(7%) | 110 (92%) | 9 (8%) |
|  | RFA | 481 | 64[5.5–72.0] | 302(63%) | 2 [1.7–2.05] | N/A | N/A | N/A | 198 (41%) | 239 (50%) | 46 (10%) | 370 (77%) | 111 (23%) |

**Supplementary Table 1**. Baseline characteristics of enrolled patients in each included study.

Data are expressed as median (range), mean ± SD or Median [IQR value]]
